# Preliminary Exploration of the Protective Mechanism of Eugenol Against Acute Liver Injury Induced by Thioacetamide Based on Metabolomics

**DOI:** 10.3390/molecules29225288

**Published:** 2024-11-08

**Authors:** Huanghan Chen, Mengting Li, Hongmu Yan, Jingyu Yan, Guang Wang, Lina Gao

**Affiliations:** 1 Department of Forensic Analytical Toxicology, School of Forensic Medicine, China Medical University, No. 77, Puhe Road, Shenyang North New Area, Shenyang 110122, China; 2 Department of Laboratory Animal Science, China Medical University, Shenyang 110122, China

**Keywords:** eugenol, metabolomics, acute liver injury, thioacetamide

## Abstract

Acute liver injury (ALI) is a significant global public health issue that can rapidly develop into acute liver failure, seriously endangering the safety of patients. Eugenol has various pharmacological effects such as antioxidant, anti-inflammatory, antibacterial, and neuroprotective properties. Through pathological section observation, eugenol can alleviate the degree of liver damage caused by thioacetamide. Based on metabolomics, a total of 87 metabolites were found to have differences in content between the control group and the thioacetamide group. Compared with the control group, the contents of 42 metabolites had increased and 45 metabolites had decreased in the thioacetamide group. These differential expressed metabolites mainly indicate inflammatory damage, oxidative damage, and abnormal energy metabolism. There were 269 metabolites with differences in content between the eugenol intervention group and the thioacetamide group. Compared with the thioacetamide group, there were 101 metabolites with increased content and 168 metabolites with decreased content in the eugenol intervention group. These differential expressed metabolites suggest that eugenol intervention can correct inflammation damage, oxidative damage, and energy metabolism abnormalities caused by TAA. This study found through pathological section observation and metabolomics that eugenol has a protective effect on acute liver injury caused by thioacetamide, and the protective mechanism may be related to the antioxidant and anti-inflammatory effects of eugenol.

## 1. Introduction

Liver disease impacts millions globally and stands as a major contributor to morbidity and mortality worldwide [1]. In China, liver diseases, predominantly drug-induced liver injury, nonalcoholic fatty liver disease, and alcoholic liver disease, afflict approximately 300 million individuals [2]. Acute liver injury denotes the abrupt harm or necrosis of liver cells resulting from a variety of factors over a brief period. It is characterized by its swift onset and diverse etiologies. Without timely intervention, the condition can progressively worsen, potentially culminating in acute liver failure at a later stage [3].

Eugenol is a simple phenolic compound characterized by the chemical formula C_10_H_12_O_2_. From a pharmacological standpoint, eugenol exhibits a range of beneficial activities, including antioxidant, anti-inflammatory, antibacterial, anesthetic, neuroprotective, antidiabetic, and anticancer properties [4,5,6,7]. Studies have demonstrated that eugenol possesses the ability to combat oxidation and neutralize reactive oxygen species (ROS). Additionally, it exerts a dose-dependent inhibitory effect on both DPPH and hydroxyl radicals [5]. It plays a significant role in delaying aging, enhancing skin health, and providing neuroprotection. Furthermore, the antioxidant properties of eugenol can also neutralize nitric oxide [6], thereby preventing the formation of reactive oxygen species (ROS) both in vivo and in vitro. It safeguards against lipid peroxidation and DNA damage induced by ROS and augments the antioxidant activity of the cellular glutathione system [8,9,10].

According to the literature, eugenol exerts its anti-inflammatory effects by inhibiting prostaglandin E (PGE) synthesis and the chemotaxis of neutrophils or macrophages. Kim et al. found in animal experiments that, at a drug dose of 160 mg/kg, alveolar collapse and multinucleated cell infiltration in experimental animal models were significantly reduced, indicating that eugenol can reduce TNF-α during lung infection and prevent neutrophil infiltration, thereby weakening the inflammatory response [11]. Additionally, eugenol can also cause dysfunction of macrophages, which balances the anti-inflammatory mediators of macrophages in the peritoneum of mice [12]. In summary, eugenol possesses pharmacological activities of anti-inflammatory and antioxidant properties.

Metabolomics is a branch of biological sciences that has emerged subsequent to genomics and proteomics. It focuses on the study of small molecule metabolites with a molecular weight below 1000 within the body, aiming to mirror the body’s condition. This field has found extensive application in pharmacological and toxicological research [13,14,15].

This study aims to employ thioacetamide in the creation of an acute liver injury model and to conduct a preliminary investigation into the protective mechanism of eugenol against acute liver injury through metabolomics, thus laying the groundwork for future clinical treatments.

## 2. Results

### 2.1. HE Staining Results

**Figure 1:**
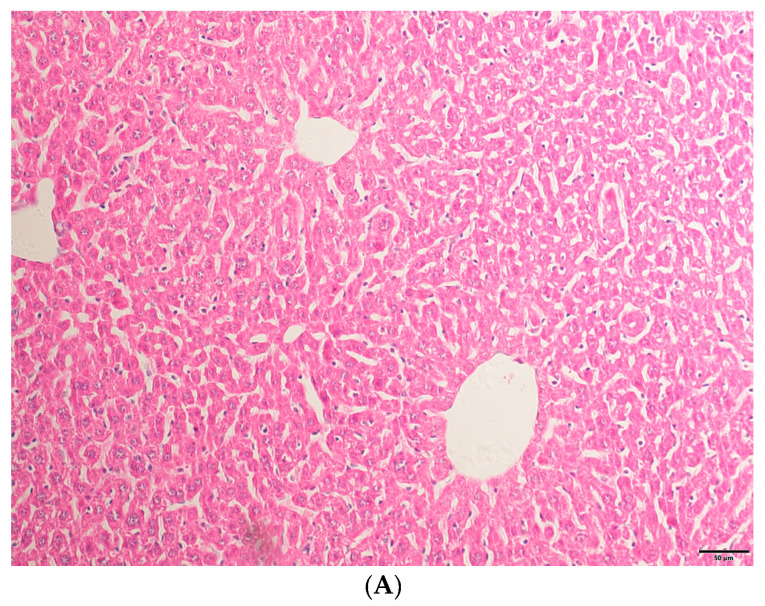

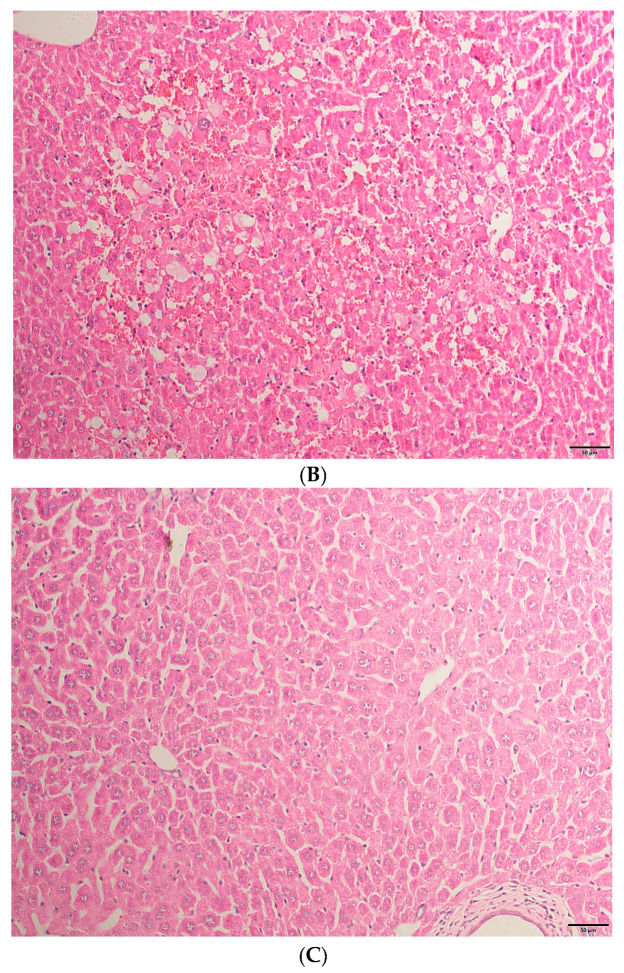
(**A**) The liver tissue structure of the NC group was basically normal. (**B**) The TAA group had disordered liver lobular structure and hepatic sinusoids congestion, accompanied by inflammatory infiltration and lipid droplet vacuolar formation. (**C**) The liver tissue structure in the eugenol treatment group was significantly improved compared to the TAA group (200× magnifications, bar = 50 µm).

Through HE staining (as shown in Figure 1), it can be found that TAA has successfully induced acute liver injury and, after intervention with eugenol, the symptoms of acute liver injury have been alleviated.

### 2.2. ALT, AST, and SOD Results

**Figure 2:**
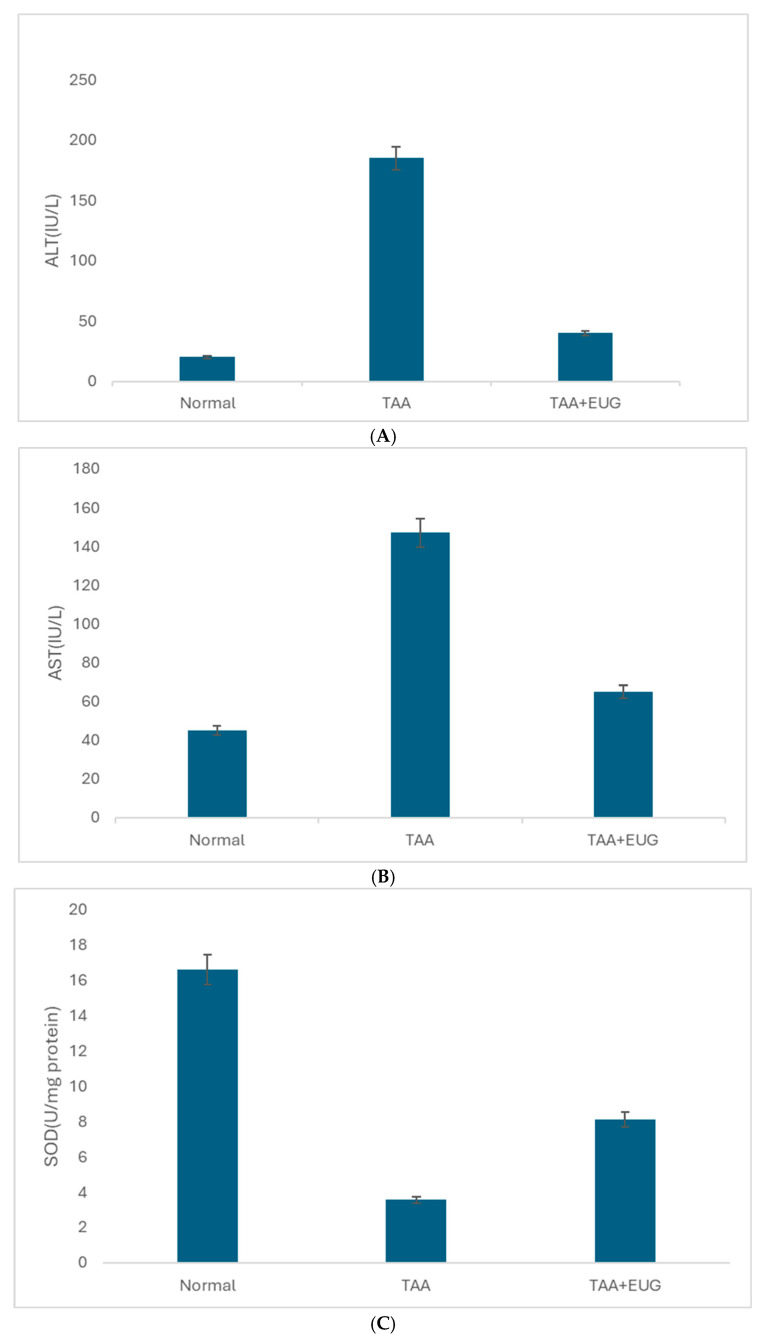
(**A**) The expression levels of ALT in the control, TAA, and EUG intervention groups; (**B**) AST in the control, TAA, and EUG intervention groups; (**C**) SOD in the control, TAA, and EUG intervention groups.

As depicted in Figure 2, in comparison to the control group, the TAA group exhibited a marked elevation in ALT and AST levels. However, subsequent to the intervention with eugenol, there was a significant reduction in both ALT and AST levels. Additionally, the SOD level in the TAA group was notably diminished relative to the control group but, after the eugenol intervention, the SOD level was significantly enhanced compared to the TAA group.

### 2.3. Metabolomics Quality Control Results

**Figure 3:**
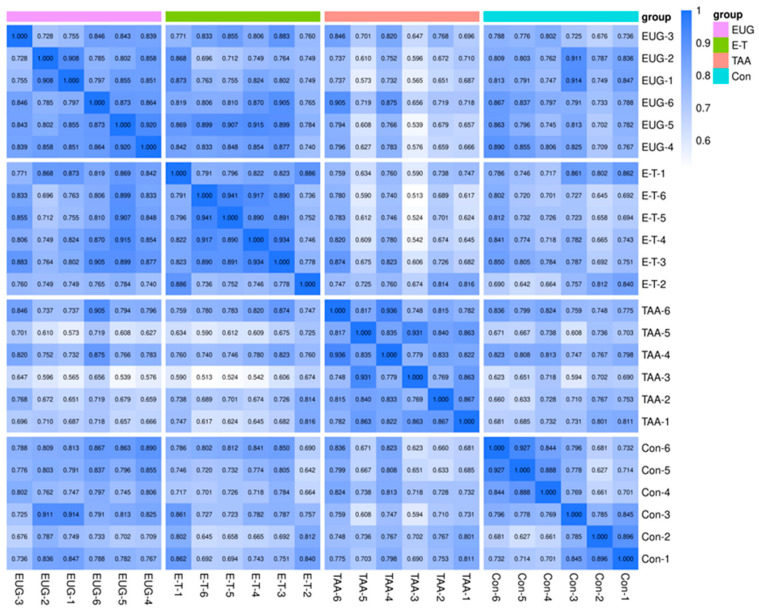
Sperman Rank Correlation across all samples. Note: the horizontal and vertical co-ordinates represent the sample name, the color depth represents the magnitude of the correlation coefficient r, and Group represents the grouping.

The correlation analysis of samples can assess the biological replicability within and between groups. A higher correlation coefficient between samples within the same group and those from different groups indicates a more reliable set of differential metabolites. Spearman Rank Correlation r is used as the metric to evaluate the correlation of biological replicates. The closer r is to 1, the stronger the correlation between the two repeated samples as shown in Figure 3.

### 2.4. PCA and OPLS-DA Results

**Figure 4:**
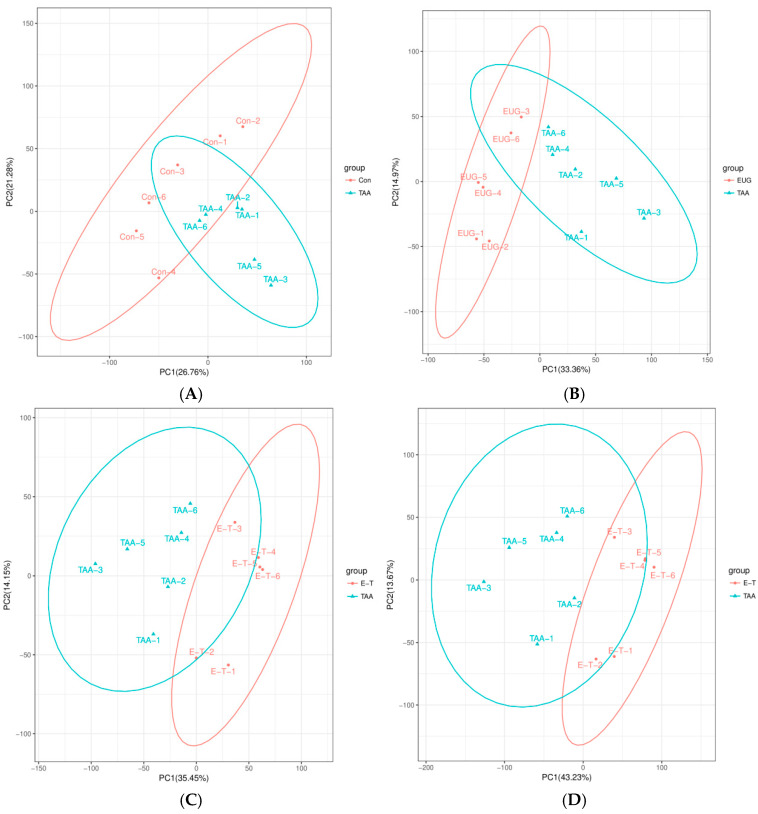
(**A**,**B**) are the PCA plots of TAA compared to the control group, where (**A**) represents in negative ion monitoring mode and (**B**) represents in positive ion monitoring mode; (**C**,**D**) is the PCA plot of the eugenol intervention group compared to the TAA group, where (**C**) represents in negative ion monitoring mode and (**D**) represents in positive ion monitoring mode.

The PCA distribution results among the various groups are depicted in Figure 4. In the graph, the horizontal axis corresponds to PC1, while the vertical axis corresponds to PC2, representing the scores of the first and second principal components, respectively. The scatter points of varying colors signify samples from distinct experimental groups, and the ellipses denote the 95% confidence intervals. The outcomes indicate that the data separation among the different groups is ideal.

**Figure 5:**
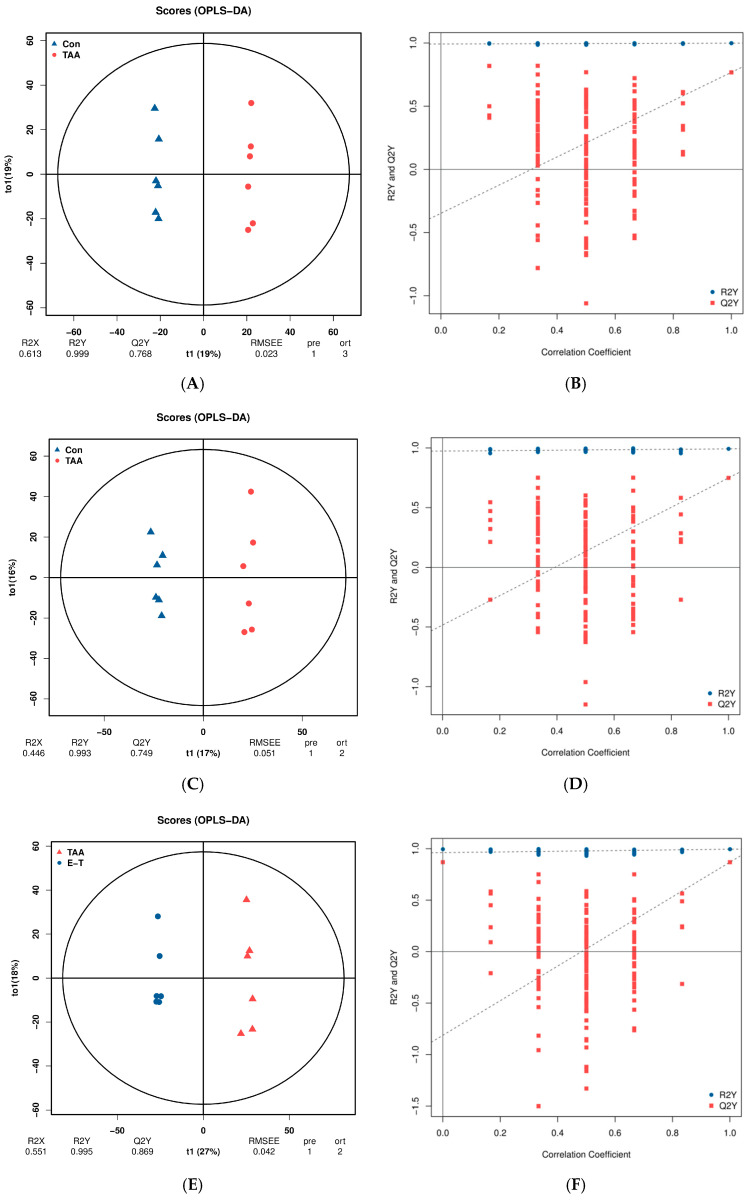

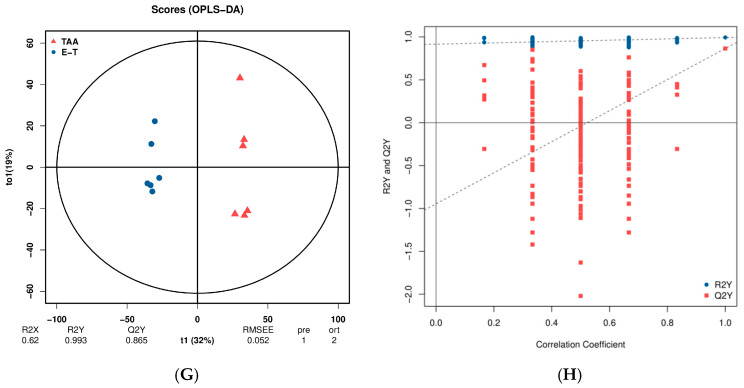
(**A**,**C**) represents OPLS-DA plot between the TAA group and the control groups; (**B**,**D**) represents OPLS-DA permutation between the TAA group and the control groups; (**E**,**G**) represents OPLS-DA plot between the TAA group and the eugenol treatment group; (**F**,**H**) represent OPLS-DA permutation between the TAA group and the eugenol intervention group; (**A**,**B**,**E**,**F**) represents in negative ion monitoring mode; (**C**,**D**,**F**,**H**) represents in positive ion monitoring mode.

Orthogonal Partial Least Squares (OPLS) distinguishes between modeling and response-related and orthogonal predictor variables based on PLS. The evaluation of Q2Y indicators and permutation testing is crucial for avoiding overfitting and assessing the statistical significance of the model. In general, a Q2Y value exceeding 0.5 indicates an effective model as shown in Figure 5.

### 2.5. Differential Expressed Metabolites

**Figure 6:**
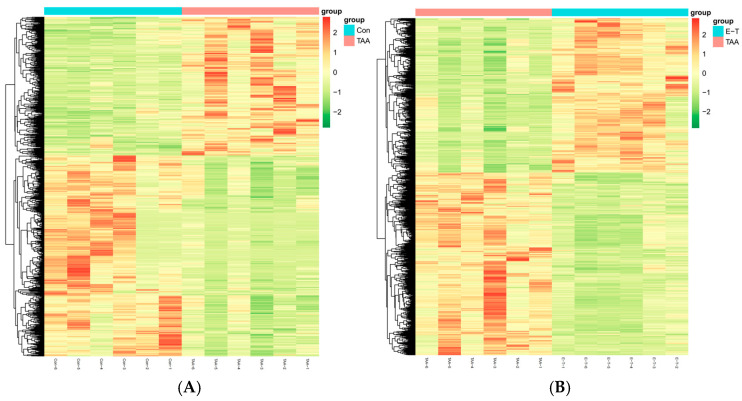
Cluster heatmap (**A**) represents the TAA group and control group; (**B**) represents the clustering diagram between TAA and eugenol intervention groups.

The cluster heatmap analysis is depicted in Figure 6. It is evident that there exists a distinct differentiation in metabolic patterns between the TAA group and the control group. Additionally, the metabolic patterns of the eugenol intervention group are also distinctly differentiated from those of the TAA group.

Compared with the control group, the differentially expressed metabolites in the TAA group are detailed in Supplementary Table S1. Utilizing metabolomics, a total of 87 metabolites were identified with significant differences in content between the control group and the thioacetamide group. Specifically, 42 metabolites exhibited increased content, while 45 metabolites showed decreased content in the thioacetamide group. Our focus is on the differentially expressed metabolites enriched by KEGG, as presented in Table 1.

After intervention with eugenol, the differential metabolites between the eugenol treatment group and the thioacetamide group are presented in Supplementary Table S2. A total of 269 metabolites exhibited differences in content between the eugenol treatment group and the thioacetamide group. Compared to the thioacetamide group, the eugenol treatment group had 101 metabolites with increased content and 168 metabolites with decreased content. The significant differential metabolites of interest in this study are detailed in Table 2.

### 2.6. Enrichment of KEGG Pathway

**Figure 7:**
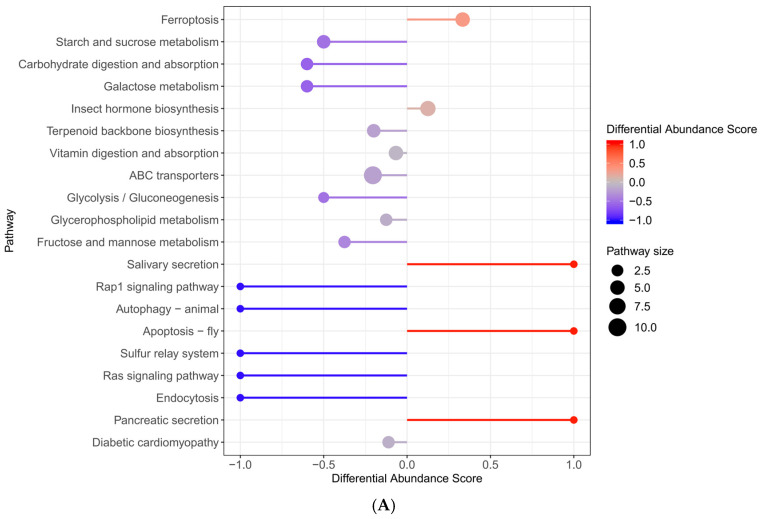

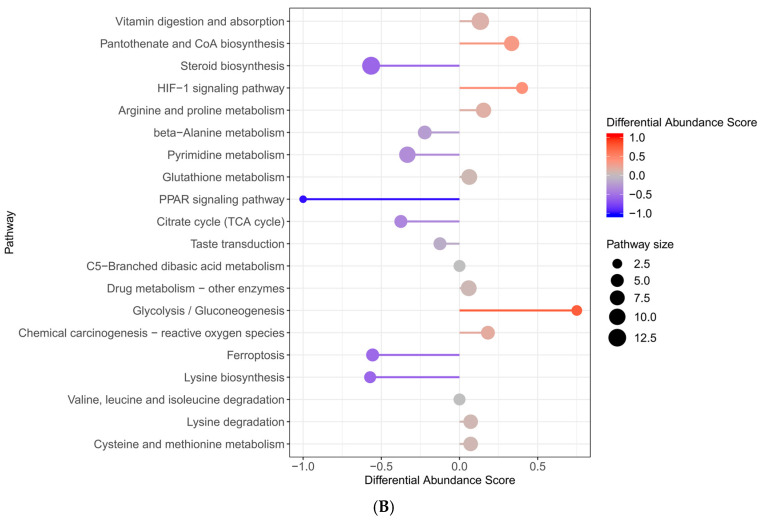
KEGG enrichment plot ((**A**) represents TAA vs. control; (**B**) represents E + T vs. TAA).

We enriched the KEGG metabolic pathway with significantly different metabolites and discovered that, as illustrated in Figure 7, the differential metabolites in the TAA groups, compared to the control group, were predominantly concentrated in energy metabolism pathways. These included pyrimidine metabolism, purine metabolism, lipid metabolism, steroid hormone biosynthesis, glycerophospholipid metabolism, ferroptosis in terms of oxidation–reduction, biosynthesis of unsaturated fatty acids, glutathione metabolism, arachidonic acid metabolism, and primary bile acid biosynthesis, which are related to the liver and gallbladder.

The distinct metabolites identified between the E + T and TAA groups were primarily associated with vitamin absorption and degradation, as well as steroid hormone biosynthesis. Regarding energy metabolism, the focus was on pyrimidine and purine metabolism. For oxidation–reduction processes, the metabolites included glutathione metabolism and unsaturated fatty acids. In terms of inflammation, arachidonic acid was a key metabolite of interest.

To assist readers in better comprehending the therapeutic effect of eugenol, Table 3 illustrates that, following eugenol intervention, the metabolite levels which underwent alterations in the TAA group exhibited a contrasting pattern of change compared to the control group.

## 3. Discussion

Acute liver injury is a common liver disease in clinical practice, often accompanied by elevated serum transaminase and bilirubin levels [16]. The liver, as the most important metabolic organ in the human body, has functions such as detoxification, coagulation, immunity, and bile secretion. It is also susceptible to stimulation by toxic chemicals, drugs, or ethanol, which can cause acute or chronic liver damage, leading to a series of metabolic diseases and even death. The severity of the condition can range from asymptomatic drug-induced elevation of liver enzymes to liver fibrosis, cirrhosis, liver cancer, and even liver failure, seriously endangering human health [17]. Currently, in clinical practice, symptomatic treatment is carried out using chemically synthesized nucleoside analogues [18], vitamin-based prophylaxis, and hepatoprotective drugs [19]. These methods can control or improve symptoms to a certain extent, but there are limitations such as insignificant therapeutic effects and side effects such as myopathy and lactic acidosis. Therefore, it is of great significance to search for novel, safe, and effective liver injury protective agents. Thioacetamide is a commonly used modeling drug for acute liver injury [20,21]. Studies have found that administering TAA to rats can cause liver damage, including inflammation, oxidative stress, and the production of profibrotic markers [22,23,24].

Metabolomics can effectively identify and quantify the levels of metabolites, often used to elucidate the pathogenesis of diseases and the mechanisms of action of drugs [25]. Through comprehensive analysis of biological samples (such as blood, urine, liver, etc.), the changes in metabolites within the body are evaluated to reflect the physiological and pathological states of the organism. This technology has been widely applied in disease diagnosis, mechanism research, and drug development [26,27]. Utilizing metabolomics, it is observed that TAA induces inflammation and oxidative stress, evidenced by a significant elevation in metabolites associated with the arachidonic acid pathway compared to the control group; metabolites linked to oxidative stress, such as an increase in oxidized glutathione, are also noted, while retinol levels decline. Additionally, there is an increase in metabolic products from the primary bile acid biosynthesis pathway and steroid hormone biosynthesis. These findings align with previously reported liver toxicity of TAA [28,29].

In the TAA group, the levels of certain substances associated with oxidation–reduction processes were observed to decrease, including alpha tocotrienol [30], retinol [31], ascorbic acid [31], and D-Octopine [32]. Conversely, the content of oxidized glutathione, 4-hydroxy-L-trhreonine, and L-4-hydroxyglutamate semialdehyde exhibited an increase, indicating oxidative damage within the TAA group. Additionally, the concentrations of inflammation-related substances such as prostaglandin D2, (15S)-15-Hydroxy-5,8,11-cis-13-trans-eicosatenoate, 20-COOH-Leukotriene B4, and 12-Keto-leukotriene B4 were found to be elevated.

Substances associated with primary bile acid biosynthesis, including 3alpha, 7alpha, 12alpha, 26-Tetrahydroxy-5beta-cholestane and 3alpha, 7alpha, 12alpha-Trihydroxy-5beta-cholestane, also experienced an increase. Conversely, the levels of unsaturated acids such as Docosenoyl CoA and Montanoyl CoA diminished. Additionally, substances linked to steroid hormone biosynthesis, such as 7alpha-Hydroxydehydroepiandrosterone and Cortolone, exhibited an increase in the TAA group.

Certain small-molecule substances associated with energy metabolism, including deoxyadenosine monophosphate, GTP, stachyose, and L-rhamnulose, were also found to be significantly different compared to the control group. Furthermore, metabolomic analysis revealed that TAA-induced liver injury is associated with ferroptosis.

Some studies have reported the anti-inflammatory properties of eugenol [6,7,33]. Through the intervention of eugenol, it was found that the content of metabolites related to arachidonic acid decreased, which is consistent with the results observed in pathological sections. Compared with the TAA group, eugenol can elevate the SOD level. Some substances that can resist oxidative stress increase, such as alpha tocotrienol, retinol, ascorbic acid, and D-octopine, which is also consistent with the antioxidant properties of eugenol [4,5,34]. The intervention of eugenol also corrected the primary bile acid biosynthesis, steroid hormone biosynthesis, and abnormal metabolism of fatty acids and energy metabolism caused by TAA. And it suggests that the protective mechanism of eugenol against acute liver injury may be mediated through the ferroptosis pathway.

## 4. Materials and Methods

### 4.1. Chemicals and Reagents

Eugenol (analytical standard, HPLC ≥ 98%) and thioacetamide reagent were purchased from Shanghai Yuanye Co., Ltd. (Shanghai, China), while formic acid, methanol, and acetonitrile were purchased from Thermo Fisher (Waltham, MA, USA). DMSO, PEG300, and Tween 80 were purchased from China National Pharmaceutical Group Chemical Reagent Co., Ltd. in Shenyang, China. The SOD assay kit, ALT, and AST assay kits were purchased from Beyotime Biotechnology Co., Ltd. (Wuhan, China).

### 4.2. Animals Grouping

(a) Preparation method of eugenol suspension: an adequate amount of eugenol was dissolved in DMSO to formulate a solution with a concentration of 150 mg/mL; subsequently, It was diluted to a concentration of 15 mg/mL using PEG300, Tween 80, and normal saline in a volume ratio of 8:1:9. A total of 0.1 mL of eugenol solution (15 mg/mL) was administered to mice at a dose of 150 mg/kg per 10 g body weight by gavage. The dosage of eugenol was selected based on literature reports [20,21]. The eugenol was administered by gavage two weeks before the administration of thioacetamide for a total of two weeks.

(b) Thioacetamide injection method: 0.1 mL of thioacetamide solution (10 mg/mL) was given by intraperitoneal injection to every 10 g body weight mice, which is a dose of 100 mg/kg per mouse [22,23]. Thioacetamide was administered intraperitoneally on the 14th day of eugenol gavage, with a total of one intraperitoneal injection. The experimental animals used in this study were all SPF-grade wild-type C57BL/6J mice (male, 10–12 weeks old, weighing 25 ± 2 g, at a total of 24, purchased from Beijing Huafukang Biotechnology Co., Ltd., Beijing, China). All experimental animals were housed in the Barrier Laboratory of the Experimental Animal Department of China Medical University, with 4–5 animals per cage. The ambient temperature was maintained at 22 ± 1 °C, and the circadian rhythm was maintained for 12 h. They were free to drink and eat. After one week of adaptive feeding, mice were randomly divided into four groups, with six mice in each group. According to the experimental protocol, they were divided into a control group, a thioacetamide model group (TAA group), and a eugenol intervention group (E + T). All experimental procedures comply with the “Regulations on the Management of Experimental Animals” issued by the National Science and Technology Commission of the People’s Republic of China and follow the “Guidelines for the Operation of Experimental Animals at China Medical University” issued by the Experimental Animal Center of China Medical University. All experiments have been approved by the Animal Ethics Committee of China Medical University (No. KT2022353). After intraperitoneal injection of thioacetamide for 24 h, all mice were humanely euthanized using compressed gas within their home cages by trained personnel. Subsequently, liver tissue and serum were collected for further testing.

### 4.3. Metabolomics

#### 4.3.1. Metabolites Extraction

A total of 50 mg of liver tissue sample was weighed, 1000 μL of extraction solution containing internal standard (2-chlorophenylalanine) (methanol/acetonitrile/water volume ratio = 2:2:1, internal standard concentration 20 mg/L) was added, vortexed, and mixed for 30 s; steel balls were added and it was treated with a 45 Hz grinder for 10 min and sonicated for 10 min (in an ice water bath); it was allowed to stand at minus 20 °C for 1 h; the sample was centrifuged at 4 °C and 12,000 rpm for 15 min; 500 μL of supernatant was carefully removed into an EP tube; the extract was dried in a vacuum concentrator; 160 μL of extraction solution (acetonitrile/water volume ratio = 1:1) was added to the dried metabolites for reconstitution; it was vortexed for 30 s and placed in an ice water bath ultrasound for 10 min; the sample was centrifuged at 4 °C and 12,000 rpm for 15 min; 120 μL of supernatant was carefully taken out and injected into a 2 mL vial. A total of 10 μL of each sample were taken and mixed to form a QC sample for machine testing. The injection volume was 1 μL.

#### 4.3.2. LC-MS/MS Analysis, Data Preprocessing

The LC/MS system employed for metabolomics is identical to that in previous studies [35]. The raw data obtained through MassLynx V4.2 are analyzed using Progenesis QI software (Version 2.0) for tasks such as peak extraction, alignment, and other processing activities. This analysis relies on the METLIN database available online via Progenesis QI for identification purposes. Additionally, both theoretical fragment identification and mass deviation are maintained within a threshold of 100 ppm.

#### 4.3.3. Data Analysis

Following the normalization of the original peak area data against the total peak area, subsequent analyses were conducted, including principal component analysis and Spearman correlation analysis. The identified compounds were examined for classification and pathway information using the KEGG, HMDB, and LipidMaps databases. Based on the classification data, we calculated and compared the difference multiples. A *t*-test was employed to assess the statistical significance (*p*-value) of each compound’s differences. Additionally, the R language package (Version 3.5.3) ropls was utilized to conduct OPLS-DA analysis, and 200 times permutation tests were performed to verify the reliability of the model. The VIP value of the model was calculated using multiple cross-validation. The method of combining the difference multiple, the *p* value, and the VIP value of the OPLS-DA model was adopted to screen the differential metabolites. The screening criteria are FC > 1, *p* value < 0.05, and VIP > 1. The different metabolites of KEGG pathway enrichment significance were calculated using a hypergeometric distribution test.

### 4.4. Hematoxylin and Eosin (H&E) Staining

Livers from the different treatment groups were fixed in 4% paraformaldehyde for 24 h; the detailed procedure was identical to that reported in our previous study [35].

### 4.5. SOD, ALT and AST

According to the kit instruction, the levels of SOD, ALT, and AST were determined in each group.

### 4.6. Statistical Analysis

Statistical analysis was conducted using SPSS 20.0 software, with normally distributed quantitative data represented as x ± s. Analysis of variance was used for overall comparison, and Student’s *t*-test was used for intergroup comparison.

## 5. Conclusions

Through this experiment, it was discovered that the mechanism of acute liver injury induced by thioacetamide is intricate, encompassing oxidative damage, inflammatory damage, and, potentially, liver fibrosis. The protective mechanism of eugenol against acute liver injury is hypothesized, based on metabolomics, to involve the reduction in oxidative and inflammatory damage, as well as the correction of aberrant energy metabolism. Nevertheless, the study’s limitation lies in its reliance on metabolomics to primarily speculate on the protective mechanism of eugenol against liver injury, while its strength is the initial exploration of the body’s holistic response. The subsequent phase involves conducting a detailed exploration at the protein and DNA levels for a specific pathway.

## Figures and Tables

**Table 1:** Differential expressed metabolites between the TAA group and control group.

No.	Name	*p* Value	VIP	Trend	KEGG_Pathway_Annotation
1	4-Hydroxy-l-threonine	0.046	1.366	up	Vitamin B6 metabolism (ko00750)
2	l-Isoleucine	0.013	1.605	down	Valine, leucine, and isoleucine degradation (ko00280); Valine, leucine, and isoleucine biosynthesis (ko00290)
3	Vitamin K1	0.002	1.931	up	Vitamin digestion and absorption (ko04977)
4	2-Phytyl-1,4-naphthoquinone	0.005	1.993	up	Ubiquinone and other terpenoid-quinone biosynthesis (ko00130); Biosynthesis of cofactors (ko01240)
5	alpha-Tocotrienol	0.039	1.548	down	Ubiquinone and other terpenoid-quinone biosynthesis (ko00130)
6	4-Amino-5-hydroxymethyl-2-methylpyrimidine	0.000	2.085	down	Thiamine metabolism (ko00730)
7	Isopentenyl phosphate	0.001	1.956	down	Terpenoid backbone biosynthesis (ko00900)
8	7alpha-Hydroxydehydroepiandrosterone	0.030	1.581	up	Steroid hormone biosynthesis (ko00140)
9	Cortolone	0.012	1.766	up	Steroid hormone biosynthesis (ko00140)
10	Retinol	0.018	1.748	down	Retinol metabolism (ko00830); Vitamin digestion and absorption (ko04977)
11	(*R*)-3-Amino-2-methylpropanoate	0.029	1.550	up	Pyrimidine metabolism (ko00240); Valine, leucine, and isoleucine degradation (ko00280)
12	GTP	0.033	1.472	down	Purine metabolism (ko00230); Riboflavin metabolism (ko00740)
13	Deoxyadenosine monophosphate	0.044	1.398	up	Purine metabolism (ko00230); Nucleotide metabolism (ko01232)
14	1-(5-Phospho-d-ribosyl)-5-amino-4-imidazolecarboxylate	0.027	1.473	up	Purine metabolism (ko00230)
15	3alpha,7alpha,12alpha,26-Tetrahydroxy-5beta-cholestane	0.007	1.881	up	Primary bile acid biosynthesis (ko00120)
16	3alpha,7alpha,12alpha-Trihydroxy-5beta-cholestane	0.034	1.477	up	Primary bile acid biosynthesis (ko00120)
17	3alpha,7alpha-Dihydroxy-5-cholestenoate	0.037	1.414	up	Primary bile acid biosynthesis (ko00120)
18	7alpha-Hydroxy-3-oxo-4-cholestenoate	0.000	2.275	up	Primary bile acid biosynthesis (ko00120)
19	Biliverdin	0.036	1.570	down	Porphyrin metabolism (ko00860); Metabolic pathways (ko01100)
20	Oxidized glutathione	0.044	1.490	up	Glutathione metabolism (ko00480); Metabolic pathways (ko01100)
21	Trypanothione	0.013	1.641	down	Glutathione metabolism (ko00480); Metabolic pathways (ko01100)
22	Stachyose	0.046	1.589	down	Galactose metabolism (ko00052); Metabolic pathways (ko01100)
23	l-Rhamnulose	0.016	1.711	down	Fructose and mannose metabolism (ko00051); Metabolic pathways (ko01100)
24	Sepiapterin	0.010	1.803	up	Folate biosynthesis (ko00790); Metabolic pathways (ko01100)
25	1-Octadecanoyl-2-(7*Z*,10*Z*,13*Z*,16*Z*-docosatetraenoyl)-sn-glycero-3-phosphoethanolamine	0.039	1.476	up	Ferroptosis (ko04216)
26	1-Octadecanoyl-sn-glycero-3-phosphoethanolamine	0.021	1.555	up	Ferroptosis (ko04216)
27	2-trans-Dodecenoyl-CoA	0.025	1.561	down	Fatty acid elongation (ko00062); Fatty acid degradation (ko00071); Metabolic pathways (ko01100); Fatty acid metabolism (ko01212)
28	trans,cis-Lauro-2,6-dienoyl-CoA	0.006	1.750	up	Fatty acid degradation (ko00071)
29	Decanoic acid	0.028	1.615	up	Fatty acid biosynthesis (ko00061); Metabolic pathways (ko01100)
30	*O*-Succinyl-l-homoserine	0.005	1.942	up	Cysteine and methionine metabolism (ko00270); Sulfur metabolism (ko00920); Metabolic pathways (ko01100); Biosynthesis of amino acids (ko01230)
31	*S*-Adenosylhomocysteine	0.047	1.540	down	Cysteine and methionine metabolism (ko00270)
32	Cyclic ADP-ribose	0.024	1.531	up	Calcium signaling pathway (ko04020)
33	Adrenic acid	0.011	1.779	up	Biosynthesis of unsaturated fatty acids (ko01040); Ferroptosis (ko04216)
34	Docosenoyl-CoA	0.040	1.587	down	Biosynthesis of unsaturated fatty acids (ko01040); Fatty acid metabolism (ko01212)
35	Montanoyl-CoA	0.021	1.777	down	Biosynthesis of unsaturated fatty acids (ko01040); Fatty acid metabolism (ko01212)
36	Threonate	0.017	1.783	up	Ascorbate and aldarate metabolism (ko00053); Metabolic pathways (ko01100)
37	Ascorbic acid	0.039	1.410	down	Ascorbate and aldarate metabolism (ko00053); Glutathione metabolism (ko00480); HIF-1 signaling pathway (ko04066); Vitamin digestion and absorption (ko04977)
38	d-Octopine	0.005	1.781	down	Arginine and proline metabolism (ko00330)
39	l-4-Hydroxyglutamate semialdehyde	0.027	1.490	up	Arginine and proline metabolism (ko00330); Metabolic pathways (ko01100)
40	20-HETE	0.022	1.751	down	Arachidonic acid metabolism (ko00590)
41	Prostaglandin D2	0.000	2.315	up	Arachidonic acid metabolism (ko00590)
42	(15S)-15-Hydroxy-5,8,11-cis-13-trans-eicosatetraenoate	0.031	1.529	up	Arachidonic acid metabolism (ko00590)
43	20-COOH-Leukotriene B4	0.040	1.527	up	Arachidonic acid metabolism (ko00590)
44	12-Keto-leukotriene B4	0.002	2.055	up	Arachidonic acid metabolism (ko00590)
45	CMP-pseudaminic acid	0.049	1.573	down	Amino sugar and nucleotide sugar metabolism (ko00520); Metabolic pathways (ko01100); Biosynthesis of nucleotide sugars (ko01250)
46	*N*-Acetylneuraminate	0.041	1.475	up	Amino sugar and nucleotide sugar metabolism (ko00520); Metabolic pathways (ko01100); Biosynthesis of nucleotide sugars (ko01250)
47	Pseudaminic acid	0.032	1.650	down	Amino sugar and nucleotide sugar metabolism (ko00520); Metabolic pathways (ko01100); Biosynthesis of nucleotide sugars (ko01250)
48	Methyl jasmonate	0.000	2.206	up	alpha-Linolenic acid metabolism (ko00592)
49	20-COOH-Leukotriene B4	0.040	1.527	up	Arachidonic acid metabolism (ko00590)

**Table 2:** Differential expressed metabolites between the E + T group and TAA group.

No	Name	*p* Value	VIP	Trend	KEGG_Pathway_Annotation
1	Vitamin K1	0.002	1.484	down	Vitamin digestion and absorption (ko04977); Ubiquinone and other terpenoid-quinone biosynthesis (ko00130)
2	Retinol	0.023	1.270	up	Vitamin digestion and absorption (ko04977); Retinol metabolism (ko00830)
3	20-HETE	0.004	1.539	up	Vascular smooth muscle contraction (ko04270); Arachidonic acid metabolism (ko00590)
4	(*R*)-3-Amino-2-methylpropanoate	0.032	1.205	down	Valine, leucine, and isoleucine degradation (ko00280); Pyrimidine metabolism (ko00240)
5	2-Phytyl-1,4-naphthoquinone	0.006	1.502	down	Ubiquinone and other terpenoid-quinone biosynthesis (ko00130)
6	alpha-Tocotrienol	0.018	1.206	up	Ubiquinone and other terpenoid-quinone biosynthesis (ko00130)
7	Isopentenyl phosphate	0.033	1.135	up	Terpenoid backbone biosynthesis (ko00900)
8	*O*-Succinyl-l-homoserine	0.005	1.481	down	Sulfur metabolism (ko00920); Cysteine and methionine metabolism (ko00270)
9	7alpha-Hydroxydehydroepiandrosterone	0.035	1.127	down	Steroid hormone biosynthesis (ko00140)
10	Cortolone	0.014	1.390	down	Steroid hormone biosynthesis (ko00140)
11	GTP	0.031	1.122	up	Rap1 signaling pathway (ko04015); Sulfur relay system (ko04122); Endocytosis (ko04144); Riboflavin metabolism (ko00740); Folate biosynthesis (ko00790); Purine metabolism (ko00230); Autophagy—animal (ko04140); Ras signaling pathway (ko04014)
12	Deoxyadenosine monophosphate	0.007	1.447	down	Purine metabolism (ko00230)
13	1-(5-Phospho-d-ribosyl)-5-amino-4-imidazolecarboxylate	0.000	1.708	down	Purine metabolism (ko00230)
14	3alpha,7alpha,12alpha,26-Tetrahydroxy-5beta-cholestane	0.002	1.467	down	Primary bile acid biosynthesis (ko00120)
15	7alpha-Hydroxy-3-oxo-4-cholestenoate	0.000	1.757	down	Primary bile acid biosynthesis (ko00120)
16	3alpha,7alpha-Dihydroxy-5beta-cholestanate	0.013	1.381	down	Primary bile acid biosynthesis (ko00120)
17	3alpha,7alpha,12alpha-Trihydroxy-5beta-cholestane	0.009	1.362	down	Primary bile acid biosynthesis (ko00120)
18	Biliverdin	0.020	1.246	up	Porphyrin metabolism (ko00860)
19	Cyclic ADP-ribose	0.037	1.112	down	Pancreatic secretion (ko04972); Calcium signaling pathway (ko04020); Oxytocin signaling pathway (ko04921); Salivary secretion (ko04970)
20	Trypanothione disulfide	0.013	1.291	up	Glutathione metabolism (ko00480)
21	Stachyose	0.024	1.256	up	Galactose metabolism (ko00052)
22	d-Fucose 6-phosphate	0.003	1.573	up	Fructose and mannose metabolism (ko00051); Amino sugar and nucleotide sugar metabolism (ko00520)
23	l-Rhamnulose	0.001	1.554	up	Fructose and mannose metabolism (ko00051)
24	Sepiapterin	0.002	1.550	down	Folate biosynthesis (ko00790)
25	1-Octadecanoyl-2-(7*Z*,10*Z*,13*Z*,16*Z*-docosatetraenoyl)-sn-glycero-3-phosphoethanolamine	0.027	1.271	down	Ferroptosis (ko04216)
26	1-Octadecanoyl-sn-glycero-3-phosphoethanolamine	0.022	1.203	down	Ferroptosis (ko04216)
27	*trans*-Tetradec-2-enoyl-CoA	0.007	1.424	up	Fatty acid elongation (ko00062); Fatty acid degradation (ko00071)
28	*trans*,*cis*-Lauro-2,6-dienoyl-CoA	0.000	1.640	down	Fatty acid degradation (ko00071)
29	Decanoic acid	0.029	1.147	down	Fatty acid biosynthesis (ko00061)
30	*S*-Adenosylhomocysteine	0.024	1.254	up	Cysteine and methionine metabolism (ko00270); Chemical carcinogenesis—reactive oxygen species (ko05208)
31	Oxidized glutathione	0.026	1.252	down	Chemical carcinogenesis—reactive oxygen species (ko05208); Glutathione metabolism (ko00480); Ferroptosis (ko04216); Thyroid hormone synthesis (ko04918); Diabetic cardiomyopathy (ko05415)
32	Adrenic acid	0.017	1.301	down	Biosynthesis of unsaturated fatty acids (ko01040); Ferroptosis (ko04216)
33	Docosenoyl-CoA	0.020	1.333	up	Biosynthesis of unsaturated fatty acids (ko01040)
34	Montanoyl-CoA	0.025	1.309	up	Biosynthesis of unsaturated fatty acids (ko01040)
35	Docosanoyl-CoA	0.024	1.300	up	Biosynthesis of unsaturated fatty acids (ko01040)
36	Prostaglandin D2	0.000	1.746	down	Asthma (ko05310); Fc epsilon RI signaling pathway (ko04664); Neuroactive ligand–receptor interaction (ko04080); Serotonergic synapse (ko04726); Arachidonic acid metabolism (ko00590); African trypanosomiasis (ko05143)
37	l-Ascorbic acid	0.036	1.099	up	Ascorbate and aldarate metabolism (ko00053); Glutathione metabolism (ko00480); Vitamin digestion and absorption (ko04977); HIF-1 signaling pathway (ko04066)
38	Threonate	0.012	1.444	down	Ascorbate and aldarate metabolism (ko00053)
39	d-Octopine	0.004	1.405	up	Arginine and proline metabolism (ko00330); ABC transporters (ko02010)
40	l-4-Hydroxyglutamate semialdehyde	0.003	1.414	down	Arginine and proline metabolism (ko00330)
41	(15*S*)-15-Hydroxy-5,8,11-*cis*-13-*trans*-eicosatetraenoate	0.041	1.118	down	Arachidonic acid metabolism (ko00590); Inflammatory mediator regulation of TRP channels (ko04750)
42	20-COOH-Leukotriene B4	0.046	1.150	down	Arachidonic acid metabolism (ko00590)
43	12-Keto-leukotriene B4	0.003	1.472	down	Arachidonic acid metabolism (ko00590)
44	CMP-pseudaminic acid	0.030	1.212	up	Amino sugar and nucleotide sugar metabolism (ko00520)
45	*N*-Acetylneuraminate	0.022	1.282	down	Amino sugar and nucleotide sugar metabolism (ko00520)
46	Pseudaminic acid	0.032	1.261	up	Amino sugar and nucleotide sugar metabolism (ko00520)
47	Methyl jasmonate	0.000	1.637	down	alpha-Linolenic acid metabolism (ko00592)
48	l-Isoleucine	0.002	1.461	up	Valine, leucine, and isoleucine biosynthesis (ko00290)
49	4-Amino-5-hydroxymethyl-2-methylpyrimidine	0.038	1.109	up	Thiamine metabolism (ko00730)

**Table 3:** The differential expressed metabolites between TAA vs. control and E + T vs. T.

No.	Name	Between the TAA Group and Control Group			Between the Eugenol Treatment Group and TAA Group			KEGG_Pathway_Annotation
		*p* Value	VIP	Trend	Trend	*p* Value	VIP	
1	4-Hydroxy-l-threonine	0.046	1.366	up	down	0.001	1.582	Vitamin B6 metabolism (ko00750)
2	l-Isoleucine	0.013	1.605	down	up	0.008	1.35	Valine, leucine, and isoleucine degradation (ko00280); Valine, leucine, and isoleucine biosynthesis (ko00290); Aminoacyl-tRNA biosynthesis (ko00970); Metabolic pathways (ko01100); 2-Oxocarboxylic acid metabolism (ko01210); Biosynthesis of amino acids (ko01230); ABC transporters (ko02010); Protein digestion and absorption (ko04974); Mineral absorption (ko04978); Shigellosis (ko05131); Central carbon metabolism in cancer (ko05230)
3	Vitamin K1	0.002	1.931	up	down	1.484	0.002	Ubiquinone and other terpenoid-quinone biosynthesis (ko00130); Metabolic pathways (ko01100); Biosynthesis of cofactors (ko01240); Vitamin digestion and absorption (ko04977)
4	2-Phytyl-1,4-naphthoquinone	0.005	1.993	up	down	0.006	1.502	Ubiquinone and other terpenoid-quinone biosynthesis (ko00130); Metabolic pathways (ko01100); Biosynthesis of cofactors (ko01240)
5	alpha-Tocotrienol	0.039	1.548	down	up	0.018	1.206	Ubiquinone and other terpenoid-quinone biosynthesis (ko00130); Metabolic pathways (ko01100)
6	4-Amino-5-hydroxymethyl-2-methylpyrimidine	0	2.085	down	up	0.002	1.461	Thiamine metabolism (ko00730); Metabolic pathways (ko01100); Biosynthesis of cofactors (ko01240); ABC transporters (ko02010)
7	Isopentenyl phosphate	0.001	1.956	down	up	0.002	1.463	Terpenoid backbone biosynthesis (ko00900); Metabolic pathways (ko01100)
8	7alpha-Hydroxydehydroepiandrosterone	0.03	1.581	up	down	0.005	1.481	Steroid hormone biosynthesis (ko00140)
9	Cortolone	0.012	1.766	up	down	0.041	1.196	Steroid hormone biosynthesis (ko00140)
10	Retinol	0.018	1.748	down	up	1.27	0.023	Retinol metabolism (ko00830); Metabolic pathways (ko01100); Biosynthesis of cofactors (ko01240); Vitamin digestion and absorption (ko04977)
11	GTP	0.033	1.472	down	up	0.021	1.265	Purine metabolism (ko00230); Riboflavin metabolism (ko00740); Folate biosynthesis (ko00790); Metabolic pathways (ko01100); Nucleotide metabolism (ko01232); Biosynthesis of cofactors (ko01240); Ras signaling pathway (ko04014); Rap1 signaling pathway (ko04015); Sulfur relay system (ko04122); Autophagy—animal (ko04140); Endocytosis (ko04144)
12	Deoxyadenosine monophosphate	0.044	1.398	up	down	0.002	1.51	Purine metabolism (ko00230); Metabolic pathways (ko01100); Nucleotide metabolism (ko01232)
13	1-(5-Phospho-d-ribosyl)-5-amino-4-imidazolecarboxylate	0.027	1.473	up	down	0.007	1.447	Purine metabolism (ko00230); Metabolic pathways (ko01100)
14	3alpha,7alpha,12alpha,26-Tetrahydroxy-5beta-cholestane	0.007	1.881	up	down	0.005	1.444	Primary bile acid biosynthesis (ko00120); Metabolic pathways (ko01100)
15	3alpha,7alpha,12alpha-Trihydroxy-5beta-cholestane	0.034	1.477	up	down	0.013	1.381	Primary bile acid biosynthesis (ko00120); Metabolic pathways (ko01100)
16	3alpha,7alpha-Dihydroxy-5-cholestenoate	0.037	1.414	up	down	0.009	1.362	Primary bile acid biosynthesis (ko00120)
17	7alpha-Hydroxy-3-oxo-4-cholestenoate	0	2.275	up	down	0.034	1.156	Primary bile acid biosynthesis (ko00120)
18	Oxidized glutathione	0.044	1.49	up	down	0.013	1.262	Glutathione metabolism (ko00480); Metabolic pathways (ko01100); Biosynthesis of cofactors (ko01240); Ferroptosis (ko04216); Thyroid hormone synthesis (ko04918); Chemical carcinogenesis—reactive oxygen species (ko05208); Diabetic cardiomyopathy (ko05415)
19	Stachyose	0.046	1.589	down	up	0.012	1.436	Galactose metabolism (ko00052); Metabolic pathways (ko01100)
20	l-Rhamnulose	0.016	1.711	down	up	0.003	1.573	Fructose and mannose metabolism (ko00051); Metabolic pathways (ko01100)
21	Sepiapterin	0.01	1.803	up	down	0.001	1.576	Folate biosynthesis (ko00790); Metabolic pathways (ko01100)
22	Adrenic acid	0.011	1.779	up	down	0.004	1.441	Biosynthesis of unsaturated fatty acids (ko01040); Ferroptosis (ko04216)
23	1-Octadecanoyl-2-(7*Z*,10*Z*,13*Z*,16*Z*-docosatetraenoyl)-sn-glycero-3-phosphoethanolamine	0.039	1.476	up	down	0.034	1.177	Ferroptosis (ko04216)
24	1-Octadecanoyl-sn-glycero-3-phosphoethanolamine	0.021	1.555	up	down	0.027	1.271	Ferroptosis (ko04216)
25	2-*trans*-Dodecenoyl-CoA	0.025	1.561	down	up	0.022	1.203	Fatty acid elongation (ko00062); Fatty acid degradation (ko00071); Metabolic pathways (ko01100); Fatty acid metabolism (ko01212)
26	trans,cis-Lauro-2,6-dienoyl-CoA	0.006	1.75	up	down	0.019	1.186	Fatty acid degradation (ko00071)
27	Decanoic acid	0.028	1.615	up	down	0	1.64	Fatty acid biosynthesis (ko00061); Metabolic pathways (ko01100)
28	*O*-Succinyl-l-homoserine	0.005	1.942	up	down	0.032	1.181	Cysteine and methionine metabolism (ko00270); Sulfur metabolism (ko00920); Metabolic pathways (ko01100); Biosynthesis of amino acids (ko01230)
29	*S*-Adenosylhomocysteine	0.047	1.54	down	up	0.002	1.471	Cysteine and methionine metabolism (ko00270); Metabolic pathways (ko01100); Biosynthesis of amino acids (ko01230); Biosynthesis of cofactors (ko01240); Chemical carcinogenesis—reactive oxygen species (ko05208)
30	Cyclic ADP-ribose	0.024	1.531	up	down	0.008	1.33	Calcium signaling pathway (ko04020); Oxytocin signaling pathway (ko04921); Salivary secretion (ko04970); Pancreatic secretion (ko04972)
31	Docosenoyl-CoA	0.04	1.587	down	up	0.013	1.266	Biosynthesis of unsaturated fatty acids (ko01040); Fatty acid metabolism (ko01212)
32	Montanoyl-CoA	0.021	1.777	down	up	0.02	1.333	Biosynthesis of unsaturated fatty acids (ko01040); Fatty acid metabolism (ko01212)
33	Ascorbic acid	0.039	1.41	down	up	0	1.746	Ascorbate and aldarate metabolism (ko00053); Glutathione metabolism (ko00480); Metabolic pathways (ko01100); Biosynthesis of cofactors (ko01240); HIF-1 signaling pathway (ko04066); Vitamin digestion and absorption (ko04977)
34	d-Octopine	0.005	1.781	down	up	0.012	1.444	Arginine and proline metabolism (ko00330); Metabolic pathways (ko01100); ABC transporters (ko02010)
35	l-4-Hydroxyglutamate semialdehyde	0.027	1.49	up	down	0.007	1.327	Arginine and proline metabolism (ko00330); Metabolic pathways (ko01100)
36	20-HETE	0.022	1.751	down	up	1.539	0.004	Arachidonic acid metabolism (ko00590); Metabolic pathways (ko01100); Vascular smooth muscle contraction (ko04270)
37	Prostaglandin D2	0	2.315	up	down	0.01	1.28	Arachidonic acid metabolism (ko00590); Metabolic pathways (ko01100); Neuroactive ligand–receptor interaction (ko04080); Fc epsilon RI signaling pathway (ko04664); Serotonergic synapse (ko04726); African trypanosomiasis (ko05143); Asthma (ko05310)
38	(15*S*)-15-Hydroxy-5,8,11-*cis*-13-*trans*-eicosatetraenoate	0.031	1.529	up	down	0.008	1.378	Arachidonic acid metabolism (ko00590); Metabolic pathways (ko01100); Inflammatory mediator regulation of TRP channels (ko04750)
39	20-COOH-Leukotriene B4	0.04	1.527	up	down	0.041	1.118	Arachidonic acid metabolism (ko00590)
40	12-Keto-leukotriene B4	0.002	2.055	up	down	0.009	1.319	Arachidonic acid metabolism (ko00590)

## Data Availability

The datasets supporting the conclusions of this article are included within the article.

## Supplementary Materials

The following supporting information can be downloaded at: https://www.mdpi.com/article/10.3390/molecules29225288/s1. Table S1. Differential expressed metabolites between the TAA group and control group. Table S2: Differential expressed metabolites between the Eugenol treatment group and TAA group.

## References

[1] Xiao J., Wang F., Wong N.K., He J., Zhang R., Sun R., Xu Y., Liu Y., Li W., Koike K. (2019). Global liver disease burdens and research trends: Analysis from a Chinese perspective. J. Hepatol..

[2] Wang F.S., Fan J.G., Zhang Z., Gao B., Wang H.Y. (2014). The global burden of liver disease: The major impact of China. Hepatology.

[3] Jaeschke H., Akakpo J.Y., Umbaugh D.S., Ramachandran A. (2020). Novel Therapeutic Approaches Against Acetaminophen-induced Liver Injury and Acute Liver Failure. Toxicol. Sci..

[4] Batiha G.E., Alkazmi L.M., Wasef L.G., Beshbishy A.M., Nadwa E.H., Rashwan E.K. (2020). *Syzygium aromaticum* L. (Myrtaceae): Traditional Uses, Bioactive Chemical Constituents, Pharmacological and Toxicological Activities. Biomolecules.

[5] Ulanowska M., Olas B. (2021). Biological Properties and Prospects for the Application of Eugenol-A Review. Int. J. Mol. Sci..

[6] Barboza J.N., da Silva Maia Bezerra Filho C., Silva R.O., Medeiros J.V.R., de Sousa D.P. (2018). An Overview on the Anti-inflammatory Potential and Antioxidant Profile of Eugenol. Oxid. Med. Cell Longev..

[7] Marchese A., Barbieri R., Coppo E., Orhan I.E., Daglia M., Nabavi S.F., Izadi M., Abdollahi M., Nabavi S.M., Ajami M. (2017). Antimicrobial activity of eugenol and essential oils containing eugenol: A mechanistic viewpoint. Crit. Rev. Microbiol..

[8] Ali S., Prasad R., Mahmood A., Routray I., Shinkafi T.S., Sahin K., Kucuk O. (2014). Eugenol-rich Fraction of *Syzygium aromaticum* (Clove) Reverses Biochemical and Histopathological Changes in Liver Cirrhosis and Inhibits Hepatic Cell Proliferation. J. Cancer Prev..

[9] Porto Mde P., da Silva G.N., Luperini B.C., Bachiega T.F., de Castro Marcondes J.P., Sforcin J.M., Salvadori D.M. (2014). Citral and eugenol modulate DNA damage and pro-inflammatory mediator genes in murine peritoneal macrophages. Mol. Biol. Rep..

[10] Kar Mahapatra S., Chakraborty S.P., Majumdar S., Bag B.G., Roy S. (2009). Eugenol protects nicotine-induced superoxide mediated oxidative damage in murine peritoneal macrophages in vitro. Eur. J. Pharmacol..

[11] Kim S.S., Oh O.J., Min H.Y., Park E.J., Kim Y., Park H.J., Nam Han Y., Lee S.K. (2003). Eugenol suppresses cyclooxygenase-2 expression in lipopolysaccharide-stimulated mouse macrophage RAW264.7 cells. Life Sci..

[12] Raghavenra H., Diwakr B.T., Lokesh B.R., Naidu K.A. (2006). Eugenol--the active principle from cloves inhibits 5-lipoxygenase activity and leukotriene-C4 in human PMNL cells. Prostagland. Leukot. Essent. Fat. Acids..

[13] Schrimpe-Rutledge A.C., Codreanu S.G., Sherrod S.D., McLean J.A. (2016). Untargeted Metabolomics Strategies-Challenges and Emerging Directions. J. Am. Soc. Mass. Spectrom..

[14] Wang R., Li B., Lam S.M., Shui G. (2020). Integration of lipidomics and metabolomics for in-depth understanding of cellular mechanism and disease progression. J. Genet. Genom..

[15] Shastry A., Dunham-Snary K. (2023). Metabolomics and mitochondrial dysfunction in cardiometabolic disease. Life Sci..

[16] Zhang F., Mao Y., Qiao H., Jiang H., Zhao H., Chen X., Tong L., Sun X. (2010). Protective effects of taurine against endotoxin-induced acute liver injury after hepatic ischemia reperfusion. Amino Acids.

[17] Chan C.C., Cheng L.Y., Lu J., Huang Y.H., Chiou S.H., Tsai P.H., Huo T.I., Lin H.C., Lee F.Y. (2012). The role of interferon-γ inducible protein-10 in a mouse model of acute liver injury post induced pluripotent stem cells transplantation. PLoS ONE.

[18] Singer G.A., Zielsdorf S., Fleetwood V.A., Alvey N., Cohen E., Eswaran S., Shah N., Chan E.Y., Hertl M., Fayek S.A. (2015). Limited hepatitis B immunoglobulin with potent nucleos(t)ide analogue is a cost effective prophylaxis against hepatitis B virus after liver transplantation. Transpl. Proc..

[19] Duan Y., Duan J., Feng Y., Huang X., Fan W., Wang K., Ouyang P., Deng Y., Du Z., Chen D. (2018). Hepatoprotective Activity of Vitamin E and Metallothionein in Cadmium-Induced Liver Injury in *Ctenopharyngodon idellus*. Oxid Med Cell Longev..

[20] Liu Z., Wang M., Wang X., Bu Q., Wang Q., Su W., Li L., Zhou H., Lu L. (2022). XBP1 deficiency promotes hepatocyte pyroptosis by impairing mitophagy to activate mtDNA-cGAS-STING signaling in macrophages during acute liver injury. Redox Biol..

[21] Elnfarawy A.A., Nashy A.E., Abozaid A.M., Komber I.F., Elweshahy R.H., Abdelrahman R.S. (2021). Vinpocetine attenuates thioacetamide-induced liver fibrosis in rats. Hum. Exp. Toxicol..

[22] Enciso N., Amiel J., Fabián-Domínguez F., Pando J., Rojas N., Cisneros-Huamaní C., Nava E., Enciso J. (2022). Model of Liver Fibrosis Induction by Thioacetamide in Rats for Regenerative Therapy Studies. Anal. Cell Pathol..

[23] Nishi K., Yagi H., Ohtomo M., Nagata S., Udagawa D., Tsuchida T., Morisaku T., Kitagawa Y. (2023). A thioacetamide-induced liver fibrosis model for pre-clinical studies in microminipig. Sci. Rep..

[24] Ezhilarasan D. (2023). Molecular mechanisms in thioacetamide-induced acute and chronic liver injury models. Env. Toxicol. Pharmacol..

[25] Ta N., Erdunduleng E., Qi R., Mu X., Feng L., Ba G., Li Y., Zhang J., Bai L., Fu M. (2023). Metabolomics analysis reveals amelioration effects of yellowhorn tea extract on hyperlipidemia, inflammation, and oxidative stress in high-fat diet-fed mice. Front. Nutr..

[26] Xiao G., Xu A., Jiang J., Chen Z., Li Y., Li S., Chen W., Zhang J., Jia C., Zeng Z. (2023). Metabolomics analysis delineates the therapeutic effects of Yinlan Tiaozhi capsule on triton WR-1339 -induced hyperlipidemia in mice. Front. Pharmacol..

[27] Luo D., Chen K., Li J., Fang Z., Pang H., Yin Y., Rong X., Guo J. (2020). Gut microbiota combined with metabolomics reveals the metabolic profile of the normal aging process and the anti-aging effect of FuFang Zhenshu TiaoZhi(FTZ) in mice. Biomed. Pharmacother..

[28] Shin M.R., Lee J.A., Kim M., Lee S., Oh M., Moon J., Nam J.W., Choi H., Mun Y.J., Roh S.S. (2021). Gardeniae Fructus Attenuates Thioacetamide-Induced Liver Fibrosis in Mice via Both AMPK/SIRT1/NF-κB Pathway and Nrf2 Signaling. Antioxidants.

[29] da Silva B.S., Paulino A.M.B., Taffarel M., Borba I.G., Telles L.O., Lima V.V., Aguiar D.H., Dias M.C., Nascimento A.F., Sinhorin V.D.G. (2021). High sucrose diet attenuates oxidative stress, inflammation and liver injury in thioacetamide-induced liver cirrhosis. Life Sci..

[30] Radhakrishnan A., Tudawe D., Chakravarthi S., Chiew G.S., Haleagrahara N. (2014). Effect of γ-tocotrienol in counteracting oxidative stress and joint damage in collagen-induced arthritis in rats. Exp. Ther. Med..

[31] Sies H. (1997). Oxidative stress: Oxidants and antioxidants. Exp. Physiol..

[32] Qu Y., Liu Z., Chen S. (2015). Study on Antioxidant Activity of Octopamine and Its Derivatives. Nat. Prod. Res. Dev..

[33] Chniguir A., Saguem M.H., Dang P.M., El-Benna J., Bachoual R. (2024). Eugenol Inhibits Neutrophils Myeloperoxidase In Vitro and Attenuates LPS-Induced Lung Inflammation in Mice. Pharmaceuticals.

[34] Bezerra D.P., Militão G.C.G., de Morais M.C., de Sousa D.P. (2017). The Dual Antioxidant/Prooxidant Effect of Eugenol and Its Action in Cancer Development and Treatment. Nutrients.

[35] Guo H., Wang G., Huang W., Li L., Bai Y., Wang H., Gao L. (2023). The Mechanism of Hepatic Encephalopathy Induced by Thioacetamide Based on Metabolomics and Proteomics: A Preliminary Study. Int. J. Mol. Sci..

