# Supplementary material for: Preliminary Exploration of the Protective Mechanism of Eugenol Against Acute Liver Injury Induced by Thioacetamide Based on Metabolomics
Source: Molecules. 2024 Nov 8;29(22):5288. doi: 10.3390/molecules29225288 (PMC11596261; doi:10.3390/molecules29225288)
Supplement: Supplementary file 1 [file molecules-29-05288-s001.zip › Supplemental Table S2.pdf]

Supplemental Table S2. Differential expressed metabolites between the Eugenol treatment group and TAA group.

name	Pvalue	VIP	regulated	KEGG_pathway_annotation
Phylloquinone	0.049707458	1.09106684	down	Vitamin digestion and absorption(ko04977);;Ubiquinone and other terpenoid-quinone biosynthesis(ko00130)
Vitamin K1	0.002497711	1.484253127	down	Vitamin digestion and absorption(ko04977);;Ubiquinone and other terpenoid-quinone biosynthesis(ko00130)
dl-alpha-Tocopherol nicotinate	0.033846313	1.139803851	down	Vitamin digestion and absorption(ko04977)
4-Hydroxy-L-threonine	0.000952083	1.581552608	down	Vitamin B6 metabolism(ko00750)
(R)-3-Amino-2-methylpropanoate	0.032459666	1.205445586	down	Valine, leucine and isoleucine degradation(ko00280);;Pyrimidine metabolism(ko00240)

3-Hydroxyisovalerate	0.000878977	1.515662788	down	Valine, leucine and isoleucine degradation(ko00280)
alpha-Isopropylmalate	0.025371715	1.185745344	down	Valine, leucine and isoleucine biosynthesis(ko00290);;Pyruvate metabolism(ko00620)
alpha-Tocopherol	0.033976584	1.183653104	down	Ubiquinone and other terpenoid-quinone biosynthesis(ko00130);;Vitamin digestion and absorption(ko04977);;Ferroptosis(ko04216)
Dihydroshikonofuran	0.001037033	1.605276657	down	Ubiquinone and other terpenoid-quinone biosynthesis(ko00130)
2-Phytyl-1,4-naphthoquinone	0.006401911	1.501869009	down	Ubiquinone and other terpenoid-quinone biosynthesis(ko00130)
beta-Tocotrienol	0.040957852	1.138503492	down	Ubiquinone and other terpenoid-quinone biosynthesis(ko00130)
Gentisate aldehyde	0.0413	1.0664	down	Tyrosine metabolism(ko00350)

	76008	07168		
gamma-Oxalocrotonate	0.018430414	1.222926172	down	Tryptophan metabolism(ko00380)
2-Aminomuconic acid	0.025361411	1.224783505	down	Tryptophan metabolism(ko00380)
4,8-Dihydroxyquinoline	0.000826556	1.511681261	down	Tryptophan metabolism(ko00380)
Oxoglutaric acid	0.001301058	1.480848141	down	Taurine and hypotaurine metabolism(ko00430);;GABAergic synapse(ko04727);;C5-Branched dibasic acid metabolism(ko00660);;Proximal tubule bicarbonate reclamation(ko04964);;Alanine, aspartate and glutamate metabolism(ko00250);;Glucagon signaling pathway(ko04922);;Pentose and glucuronate interconversions(ko00040);;Bile secretion(ko04976);;Butanoate metabolism(ko00650);;D-Amino acid metabolism(ko00470);;HIF-1 signaling pathway(ko04066);;Ascorbate and aldarate metabolism(ko00053);;Glyoxylate and dicarboxylate metabolism(ko00630);;Arginine biosynthesis(ko00220);;Central carbon metabolism in cancer(ko05230);;Histidine metabolism(ko00340);;Citrate cycle (TCA cycle)(ko00020);;Lysine degradation(ko00310);;Lysine biosynthesis(ko00300)
L-Malic acid	0.0	1.1	do	Taste transduction(ko04742);;Glucagon signaling pathway(ko04922);;Citrate cycle

	37542596	35769037	wn	(TCA cycle)(ko00020);;Pathways in cancer(ko05200);;Renal cell carcinoma(ko05211);;Pyruvate metabolism(ko00620);;Central carbon metabolism in cancer(ko05230);;Proximal tubule bicarbonate reclamation(ko04964);;Glyoxylate and dicarboxylate metabolism(ko00630)
L-Homocysteine	0.031513514	1.181139086	down	Sulfur metabolism(ko00920);;Cysteine and methionine metabolism(ko00270);;NOD-like receptor signaling pathway(ko04621);;Antifolate resistance(ko01523)
O-Succinyl-L-homoserine	0.005435108	1.480788169	down	Sulfur metabolism(ko00920);;Cysteine and methionine metabolism(ko00270)
7alpha-Hydroxydehydroepiandrosterone	0.034705448	1.127284886	down	Steroid hormone biosynthesis(ko00140)
Cholesterol sulfate	0.028784385	1.107737881	down	Steroid hormone biosynthesis(ko00140)
22(R)-Hydroxycholesterol	0.04874375	1.027466964	down	Steroid hormone biosynthesis(ko00140)
3alpha,11beta,21-Trihydroxy-20-oxo-5beta-pregnan-18-al	0.0409	1.1957	down	Steroid hormone biosynthesis(ko00140)

	41401	44005		
Cortolone	0.01396101	1.389606847	down	Steroid hormone biosynthesis(ko00140)
Androsterone	0.005897031	1.385628062	down	Steroid hormone biosynthesis(ko00140)
11-Dehydrocorticosterone	0.013092518	1.352259772	down	Steroid hormone biosynthesis(ko00140)
4-Methylpentanal	0.032551456	1.195888868	down	Steroid hormone biosynthesis(ko00140)
Pregnanediol	0.00794419	1.332492781	down	Steroid hormone biosynthesis(ko00140)
3beta-Hydroxypregn-5-en-20-one sulfate	0.005841531	1.449928251	down	Steroid hormone biosynthesis(ko00140)

Calcidiol	0.00352098	1.422556071	down	Steroid biosynthesis(ko00100);;Tuberculosis(ko05152);;Parathyroid hormone synthesis, secretion and action(ko04928)
Calcitetrol	0.044897044	1.147868169	down	Steroid biosynthesis(ko00100);;Parathyroid hormone synthesis, secretion and action(ko04928)
7-dehydrocholesterol	0.012626818	1.303032955	down	Steroid biosynthesis(ko00100);;Insect hormone biosynthesis(ko00981)
Ergosta-5,7,22,24(28)-tetraen-3beta-ol	0.017251744	1.19505335	down	Steroid biosynthesis(ko00100)
24-Ethylidenelophenol	0.011925892	1.323682331	down	Steroid biosynthesis(ko00100)
Ergosterol	0.000911671	1.545796844	down	Steroid biosynthesis(ko00100)
5-Dehydroepisterol	0.0017	1.4887	down	Steroid biosynthesis(ko00100)

	90419	87013		
4alpha-Methylzymosterol	0.040034923	1.09157675	down	Steroid biosynthesis(ko00100)
4alpha-Methyl-5alpha-ergosta-8,14,24(28)-trien-3beta-ol	0.002919515	1.424751463	down	Steroid biosynthesis(ko00100)
Campesterol	0.006007615	1.434890182	down	Steroid biosynthesis(ko00100)
Stigmasterol	0.019960296	1.213964261	down	Steroid biosynthesis(ko00100)
4alpha-carboxy-4beta-methyl-5alpha-cholesta-8,24-dien-3beta-ol	0.010307117	1.414833995	down	Steroid biosynthesis(ko00100)
3-Ketosucrose	0.000712175	1.523961969	down	Starch and sucrose metabolism(ko00500)

5-Amino-6-(5'-phosphoribosylamino)uracil	0.0213703 7	1.2596184 89	down	Riboflavin metabolism(ko00740)
all-trans-3,4-Didehydroretinol	0.0014088 61	1.5974062 84	down	Retinol metabolism(ko00830)
ADP	0.0210441 31	1.2647417 58	down	Renin secretion(ko04924);;Taste transduction(ko04742);;Purine metabolism(ko00230);;Lysosome(ko04142);;AMPK signaling pathway(ko04152);;Thermogenesis(ko04714);;Oxidative phosphorylation(ko00190);;FoxO signaling pathway(ko04068);;Parkinson disease(ko05012);;Pathways of neurodegeneration - multiple diseases(ko05022);;Neuroactive ligand-receptor interaction(ko04080);;Platelet activation(ko04611);;Amyotrophic lateral sclerosis(ko05014)
(R)-S-Lactoylglutathione	0.0279631 28	1.1405095 03	down	Pyruvate metabolism(ko00620)
2'-Deoxy-5-hydroxymethylcytidine-5'-triphosphate	0.0114217 34	1.2983564 7	down	Pyrimidine metabolism(ko00240)
dCDP	0.0050164	1.3748185	down	Pyrimidine metabolism(ko00240)

	38	65		
CMP	0.035876334	1.118930558	down	Pyrimidine metabolism(ko00240)
Thymidine	0.040500232	1.094709178	down	Pyrimidine metabolism(ko00240)
(Z)-3-Ureidoacrylate peracid	0.031089365	1.402032691	down	Pyrimidine metabolism(ko00240)
5-Methylcytosine	0.012766744	1.476039047	down	Pyrimidine metabolism(ko00240)
(R)(-)-Allantoin	0.027120443	1.258135869	down	Purine metabolism(ko00230)
N-Formiminoglycine	0.004215008	1.435093631	down	Purine metabolism(ko00230)
dGDP	0.0	1.4	do	Purine metabolism(ko00230)

	01921366	92297583	wn	
dGMP	0.00222678	1.509956458	down	Purine metabolism(ko00230)
Deoxyadenosine monophosphate	0.00675122	1.447317049	down	Purine metabolism(ko00230)
1-(5-Phospho-D-ribosyl)-5-amino-4-imidazolecarboxylate	0.000269249	1.708402451	down	Purine metabolism(ko00230)
5-Amino-4-imidazole carboxylate	0.020723037	1.22400528	down	Purine metabolism(ko00230)
Butyric acid	0.024377447	1.187291534	down	Protein digestion and absorption(ko04974);;Carbohydrate digestion and absorption(ko04973);;Butanoate metabolism(ko00650)
p-cresol	6.87E-05	1.6554837	down	Protein digestion and absorption(ko04974)

		08		
Androstenedione	0.005152663	1.444414653	down	Prostate cancer(ko05215);;Ovarian steroidogenesis(ko04913);;Endocrine resistance(ko01522);;Steroid hormone biosynthesis(ko00140);;Prolactin signaling pathway(ko04917);;Pathways in cancer(ko05200)
3alpha,7alpha,12alpha,26-Tetrahydroxy-5beta-cholestane	0.002272358	1.466884957	down	Primary bile acid biosynthesis(ko00120)
4-Cholesten-7alpha,12alpha-diol-3-one	0.034174569	1.155986488	down	Primary bile acid biosynthesis(ko00120)
7alpha-Hydroxy-3-oxo-4-cholestenoate	4.03E-05	1.756644752	down	Primary bile acid biosynthesis(ko00120)
3alpha,7alpha-Dihydroxy-5beta-cholestanate	0.012603831	1.380562979	down	Primary bile acid biosynthesis(ko00120)
3alpha,7alpha,12alpha-Trihydroxy-5beta-cholestane	0.008578304	1.361720238	down	Primary bile acid biosynthesis(ko00120)
7alpha,12alpha-Dihydroxy-5beta	0.0	1.0	do	Primary bile acid biosynthesis(ko00120)

-cholestan-3-one	3834434	91461061	wn	
8(S)-HETE	0.001167015	1.488309425	down	PPAR signaling pathway(ko03320);;Arachidonic acid metabolism(ko00590)
Biliverdin-IX-delta	0.049311681	1.204519105	down	Porphyrin metabolism(ko00860)
3-Hydroxyethylchlorophyllide a	0.009928014	1.301105557	down	Porphyrin metabolism(ko00860)
5-Oxo-delta-bilirubin	0.000998471	1.554039678	down	Porphyrin metabolism(ko00860)
Biliverdin-IX-beta	0.016262234	1.404106731	down	Porphyrin metabolism(ko00860)
N-Acetylbialaphos	0.0116320	1.3972310	down	Phosphonate and phosphinate metabolism(ko00440)

	11	76		
Demethylphosphinothricin	0.0<br>140<br>779<br>17	1.2<br>967<br>350<br>41	do<br>wn	Phosphonate and phosphinate metabolism(ko00440)
Chorismate	0.0<br>001<br>478<br>37	1.6<br>981<br>903<br>4	do<br>wn	Phenylalanine, tyrosine and tryptophan biosynthesis(ko00400);;Ubiquinone and other terpenoid-quinone biosynthesis(ko00130);;Folate biosynthesis(ko00790)
L-quinate	0.0<br>016<br>272<br>74	1.4<br>669<br>131<br>69	do<br>wn	Phenylalanine, tyrosine and tryptophan biosynthesis(ko00400)
Phenylacetaldehyde	0.0<br>017<br>011<br>11	1.4<br>602<br>742<br>63	do<br>wn	Phenylalanine metabolism(ko00360)
6-Aminopenicillanate	0.0<br>397<br>724<br>65	1.1<br>112<br>119<br>42	do<br>wn	Penicillin and cephalosporin biosynthesis(ko00311)
Phosphoric acid	0.0<br>106<br>630<br>16	1.2<br>838<br>383<br>8	do<br>wn	Pathways of neurodegeneration - multiple diseases(ko05022);;Parkinson disease(ko05012);;Oxidative phosphorylation(ko00190);;Mineral absorption(ko04978);;ABC transporters(ko02010);;Parathyroid hormone synthesis, secretion and action(ko04928)
Pyrophosphate	0.0	1.2	do	Pathways of neurodegeneration - multiple diseases(ko05022);;Oxidative

	13847182	56490807	wn	phosphorylation(ko00190);;Parkinson disease(ko05012)
Dopamine quinone	0.001002187	1.53342077	down	Parkinson disease(ko05012)
(R)-4-Dehydropantoate	0.008372964	1.3304301	down	Pantothenate and CoA biosynthesis(ko00770)
Cyclic ADP-ribose	0.036777149	1.112424405	down	Pancreatic secretion(ko04972);;Calcium signaling pathway(ko04020);;Oxytocin signaling pathway(ko04921);;Salivary secretion(ko04970)
Carbamoyl phosphate	0.00172085	1.451775106	down	Nitrogen metabolism(ko00910);;Purine metabolism(ko00230);;Arginine biosynthesis(ko00220);;Pyrimidine metabolism(ko00240);;Alanine, aspartate and glutamate metabolism(ko00250)
Nicotine	0.01888669	1.249090401	down	Nicotine addiction(ko05033);;Thermogenesis(ko04714);;Metabolism of xenobiotics by cytochrome P450(ko00980);;Chemical carcinogenesis - receptor activation(ko05207)
5-(N-Methyl-4,5-dihydro-1H-pyrrol-2-yl)pyridin-2-ol	0.0389758	1.2347737	down	Nicotinate and nicotinamide metabolism(ko00760)

	46	46		
1-Nitro-7-glutathionyl-8-hydroxy-7,8-dihydronaphthalene	0.000199954	1.607152002	down	Metabolism of xenobiotics by cytochrome P450(ko00980)
S-[2-(N7-Guanyl)ethyl]-N-acetyl-L-cysteine	0.015940035	1.255035066	down	Metabolism of xenobiotics by cytochrome P450(ko00980)
Bromobenzene-2,3-oxide	0.041892331	1.106708992	down	Metabolism of xenobiotics by cytochrome P450(ko00980)
6-[2,3-Dihydroxy-1-(hydroxymethyl)propyl]-1,2-dihydro-7-hydroxy-9-methoxy-cyclopenta[c][1]benzopyran-3,4-dione	0.00504022	1.446710878	down	Metabolism of xenobiotics by cytochrome P450(ko00980)
N6,N6,N6-Trimethyl-L-lysine	0.005512086	1.400190263	down	Lysine degradation(ko00310)
L-Pipecolate	0.002301606	1.440179712	down	Lysine degradation(ko00310)
(2R,3R)-3-Methylornithinyl-N6-	0.0	1.4	do	Lysine biosynthesis(ko00300)

lysine	03810179	18301447	wn	
(Z)-But-1-ene-1,2,4-tricarboxylate	6.28E-05	1.741015076	down	Lysine biosynthesis(ko00300)
Sirolimus	0.032968915	1.118117543	down	Longevity regulating pathway - worm(ko04212);;Cellular senescence(ko04218);;Longevity regulating pathway(ko04211)
Delta4-Dafachronic acid	0.03124883	1.146888933	down	Longevity regulating pathway - worm(ko04212)
Dihydrolipoate	0.003029255	1.584324602	down	Lipoic acid metabolism(ko00785)
13(S)-HODE	0.040672782	1.077335789	down	Linoleic acid metabolism(ko00591);;PPAR signaling pathway(ko03320)
Dihomo-gamma-linolenate	0.0033623	1.3693165	down	Linoleic acid metabolism(ko00591);;Biosynthesis of unsaturated fatty acids(ko01040)

	99	46		
9,10,13-TriHOME	0.005652245	1.432192322	down	Linoleic acid metabolism(ko00591)
9,12,13-TriHOME	0.009324621	1.301812704	down	Linoleic acid metabolism(ko00591)
9,10-Epoxy-13-hydroxy-11-octadecenoate	0.002179933	1.449649804	down	Linoleic acid metabolism(ko00591)
(+)-Camphor	0.019582851	1.252800467	down	Inflammatory mediator regulation of TRP channels(ko04750)
Anserine	0.004688921	1.486719966	down	Histidine metabolism(ko00340);;beta-Alanine metabolism(ko00410)
Dihydrourocanate	0.008444473	1.321657406	down	Histidine metabolism(ko00340)
L-Histidinol	0.0	1.4	do	Histidine metabolism(ko00340)

	01822132	73382355	wn	
4-Hydroxynonenal	0.005459699	1.347889679	down	Hepatocellular carcinoma(ko05225);;Pathways in cancer(ko05200)
Citric acid	0.017682652	1.282008741	down	Glyoxylate and dicarboxylate metabolism(ko00630);;Alanine, aspartate and glutamate metabolism(ko00250);;Central carbon metabolism in cancer(ko05230);;Glucagon signaling pathway(ko04922);;Citrate cycle (TCA cycle)(ko00020);;Taste transduction(ko04742)
4-Hydroxy-2-oxoglutaric acid	0.011569045	1.35334404	down	Glyoxylate and dicarboxylate metabolism(ko00630)
Pyroglutamic acid	0.008696762	1.300389938	down	Glutathione metabolism(ko00480)
Glutathionylspermine	0.011742987	1.435826251	down	Glutathione metabolism(ko00480)
L-Fucono-1,5-lactone	0.0005251	1.5759610	down	Fructose and mannose metabolism(ko00051)

	55	52		
Sepiapterin	0.001520765	1.549709112	down	Folate biosynthesis(ko00790)
Neopterin	0.00206874	1.447668949	down	Folate biosynthesis(ko00790)
Tetrahydrobiopterin	0.034069866	1.176794754	down	Fluid shear stress and atherosclerosis(ko05418);;Folate biosynthesis(ko00790)
1-Octadecanoyl-2-(7Z,10Z,13Z,16Z-docosatetraenoyl)-sn-glycero-3-phosphoethanolamine	0.026938735	1.270843166	down	Ferroptosis(ko04216)
1-Octadecanoyl-sn-glycero-3-phosphoethanolamine	0.021918897	1.203192356	down	Ferroptosis(ko04216)
trans,cis-Lauro-2,6-dienoyl-CoA	0.000120337	1.639748148	down	Fatty acid degradation(ko00071)
Decanoic acid	0.0	1.1	do	Fatty acid biosynthesis(ko00061)

	28760539	47077776	wn	
(9Z)-Hexadecenoic acid	0.04967337	1.030687612	down	Fatty acid biosynthesis(ko00061)
5-Fluorodeoxyuridine	0.046660467	1.163808551	down	Drug metabolism - other enzymes(ko00983)
alpha-Fluoro-beta-ureidopropionic acid	0.008241891	1.342966469	down	Drug metabolism - other enzymes(ko00983)
Fluoroacetic acid	0.005278706	1.425022844	down	Drug metabolism - other enzymes(ko00983)
Carmofur	0.003005594	1.487628494	down	Drug metabolism - other enzymes(ko00983)
Endoxifen	0.0196414	1.3272837	down	Drug metabolism - cytochrome P450(ko00982);;Endocrine resistance(ko01522)

	46	65		
S-Adenosyl-L-homocysteine	0.001613324	1.471244937	down	Cysteine and methionine metabolism(ko00270);;Chemical carcinogenesis - reactive oxygen species(ko05208)
Behenic acid	0.029832739	1.154469586	down	Cutin, suberine and wax biosynthesis(ko00073);;Biosynthesis of unsaturated fatty acids(ko01040)
22-Hydroxydocosanoic acid	0.003946744	1.422971529	down	Cutin, suberine and wax biosynthesis(ko00073)
17alpha-Hydroxypregnenolone	0.016339647	1.231568264	down	Cushing syndrome(ko04934);;Steroid hormone biosynthesis(ko00140);;Cortisol synthesis and secretion(ko04927);;Ovarian steroidogenesis(ko04913)
Cholesterol	0.001835672	1.479145293	down	Cushing syndrome(ko04934);;Lipid and atherosclerosis(ko05417);;Pathways in cancer(ko05200);;Cholesterol metabolism(ko04979);;Steroid biosynthesis(ko00100);;Steroid hormone biosynthesis(ko00140);;Bile secretion(ko04976);;Primary bile acid biosynthesis(ko00120);;Ovarian steroidogenesis(ko04913);;Cortisol synthesis and secretion(ko04927);;Insect hormone biosynthesis(ko00981);;Basal cell carcinoma(ko05217);;Aldosterone synthesis and secretion(ko04925);;Vitamin digestion and absorption(ko04977);;Fat digestion and absorption(ko04975)
Pregnenolone	0.0	1.5	do	Cortisol synthesis and secretion(ko04927);;Ovarian

	02476941	26132913	wn	steroidogenesis(ko04913);;Steroid hormone biosynthesis(ko00140);;Aldosterone synthesis and secretion(ko04925);;Cushing syndrome(ko04934)
Oxidized glutathione	0.026359621	1.251975285	down	Chemical carcinogenesis - reactive oxygen species(ko05208);;Glutathione metabolism(ko00480);;Ferroptosis(ko04216);;Thyroid hormone synthesis(ko04918);;Diabetic cardiomyopathy(ko05415)
L-Asparagine	0.027135317	1.151253534	down	Central carbon metabolism in cancer(ko05230);;Aminoacyl-tRNA biosynthesis(ko00970);;Alanine, aspartate and glutamate metabolism(ko00250);;Protein digestion and absorption(ko04974);;Mineral absorption(ko04978)
cis-Aconitic acid	0.027653347	1.24455503	down	C5-Branched dibasic acid metabolism(ko00660);;Glyoxylate and dicarboxylate metabolism(ko00630);;Citrate cycle (TCA cycle)(ko00020)
4-Hydroxybutanoic acid	0.002186151	1.456089837	down	Butanoate metabolism(ko00650)
KAPA	0.012869963	1.296539092	down	Biotin metabolism(ko00780)
8-Amino-7-oxononanoate	0.0036608	1.5030599	down	Biotin metabolism(ko00780)

	77	58		
7,8-Diaminononanoate	0.001964489	1.473377635	down	Biotin metabolism(ko00780)
8-Amino-7-oxononanoic acid	0.004309825	1.441165983	down	Biotin metabolism(ko00780)
Adrenic acid	0.016562229	1.30095578	down	Biosynthesis of unsaturated fatty acids(ko01040);;Ferroptosis(ko04216)
(9Z)-Octadecenoic acid	0.002424027	1.484729424	down	Biosynthesis of unsaturated fatty acids(ko01040);;Cutin, suberine and wax biosynthesis(ko00073);;Fatty acid biosynthesis(ko00061);;Longevity regulating pathway - worm(ko04212)
Docosanoic acid	0.012954101	1.266474855	down	Biosynthesis of unsaturated fatty acids(ko01040);;Cutin, suberine and wax biosynthesis(ko00073)
Docosahexaenoic acid	0.020059262	1.249913277	down	Biosynthesis of unsaturated fatty acids(ko01040)
Tetracosanoic acid	0.0	1.1	do	Biosynthesis of unsaturated fatty acids(ko01040)

	44475859	44171459	wn	
Spermine	0.012577666	1.2688678	down	Bile secretion(ko04976);;Arginine and proline metabolism(ko00330);;Pantothenate and CoA biosynthesis(ko00770);;beta-Alanine metabolism(ko00410);;Glutathione metabolism(ko00480)
Rifampicin	0.034050814	1.138359705	down	Bile secretion(ko04976)
L-Histidine	0.022919946	1.222376367	down	beta-Alanine metabolism(ko00410);;Histidine metabolism(ko00340);;Protein digestion and absorption(ko04974);;D-Amino acid metabolism(ko00470);;ABC transporters(ko02010);;Aminoacyl-tRNA biosynthesis(ko00970);;Central carbon metabolism in cancer(ko05230)
beta-Alanyl-L-arginine	0.022089453	1.275225809	down	beta-Alanine metabolism(ko00410)
Prostaglandin D2	4.02E-06	1.745509368	down	Asthma(ko05310);;Fc epsilon RI signaling pathway(ko04664);;Neuroactive ligand-receptor interaction(ko04080);;Serotonergic synapse(ko04726);;Arachidonic acid metabolism(ko00590);;African trypanosomiasis(ko05143)
L-Arabinono-1,4-lactone	0.0145861	1.2600728	down	Ascorbate and aldarate metabolism(ko00053)

	3	48		
Threonate	0.011666376	1.443598118	down	Ascorbate and aldarate metabolism(ko00053)
Feruloylputrescine	0.007303058	1.326993994	down	Arginine and proline metabolism(ko00330)
L-4-Hydroxyglutamate semialdehyde	0.003082068	1.414024764	down	Arginine and proline metabolism(ko00330)
Prostaglandin G2	0.007717893	1.378137358	down	Arachidonic acid metabolism(ko00590);;Serotonergic synapse(ko04726);;Platelet activation(ko04611)
(15S)-15-Hydroxy-5,8,11-cis-13-trans-eicosatetraenoate	0.04051805	1.117874495	down	Arachidonic acid metabolism(ko00590);;Inflammatory mediator regulation of TRP channels(ko04750)
20-COOH-Leukotriene B4	0.045998707	1.150348008	down	Arachidonic acid metabolism(ko00590)
16(R)-HETE	0.0	1.3	do	Arachidonic acid metabolism(ko00590)

	09124452	18962185	wn	
12-Keto-leukotriene B4	0.003323692	1.471928582	down	Arachidonic acid metabolism(ko00590)
Sphingosine 1-phosphate	0.003671735	1.567326769	down	Apelin signaling pathway(ko04371);;Neuroactive ligand-receptor interaction(ko04080);;Sphingolipid metabolism(ko00600);;Phospholipase D signaling pathway(ko04072);;Tuberculosis(ko05152);;Fc gamma R-mediated phagocytosis(ko04666);;Calcium signaling pathway(ko04020);;Sphingolipid signaling pathway(ko04071)
L-Pyrrolysine	0.011637258	1.29423527	down	Aminoacyl-tRNA biosynthesis(ko00970);;Lysine biosynthesis(ko00300);;Protein digestion and absorption(ko04974)
N-Acetylneuraminate	0.021556583	1.28164344	down	Amino sugar and nucleotide sugar metabolism(ko00520)
D-Glucosamine	0.000321334	1.596258508	down	Amino sugar and nucleotide sugar metabolism(ko00520)
2(R)-HOT	0.0043	1.4424	down	alpha-Linolenic acid metabolism(ko00592)

	99826	43773		
9-Hydroxy-12-oxo-15(Z)-octadecenoic acid	0.00333883	1.419673051	down	alpha-Linolenic acid metabolism(ko00592)
Methyl jasmonate	0.00013361	1.637150126	down	alpha-Linolenic acid metabolism(ko00592)
Isoproterenol	0.008333105	1.349589037	down	Adrenergic signaling in cardiomyocytes(ko04261)
Norfloxacin	0.009937143	1.333124934	down	ABC transporters(ko02010)
Retinol	0.022748475	1.270179771	up	Vitamin digestion and absorption(ko04977);;Retinol metabolism(ko00830)
Vitamin A	0.045755897	1.091223109	up	Vitamin digestion and absorption(ko04977);;Retinol metabolism(ko00830)

20-HETE	0.0038921 29	1.5390431 41	up	Vascular smooth muscle contraction(ko04270);;Arachidonic acid metabolism(ko00590)
Acetoacetate	0.0133541 44	1.3931761 45	up	Valine, leucine and isoleucine degradation(ko00280);;Lysine degradation(ko00310);;Tyrosine metabolism(ko00350);;Butanoate metabolism(ko00650)
trans-Cinnamic acid	0.0025792 95	1.4200649 55	up	Ubiquinone and other terpenoid-quinone biosynthesis(ko00130);;Phenylalanine metabolism(ko00360)
3''-Hydroxy-geranylhydroquinone	0.0131362 17	1.2287461 37	up	Ubiquinone and other terpenoid-quinone biosynthesis(ko00130)
alpha-Tocotrienol	0.0177733 05	1.2058692 69	up	Ubiquinone and other terpenoid-quinone biosynthesis(ko00130)
Phenol	0.0269763 34	1.1576626 38	up	Tyrosine metabolism(ko00350);;Chemical carcinogenesis - reactive oxygen species(ko05208);;Protein digestion and absorption(ko04974)
N-Acetyl-5-hydroxytryptamine	0.0019	1.4629	up	Tryptophan metabolism(ko00380)

	27495	45749		
Isopentenyl phosphate	0.032567496	1.134924875	up	Terpenoid backbone biosynthesis(ko00900)
Salicin	0.001325834	1.524601026	up	Taste transduction(ko04742);;Glycolysis / Gluconeogenesis(ko00010)
2-Methoxy-estradiol-17beta 3-glucuronide	0.000447553	1.610460496	up	Steroid hormone biosynthesis(ko00140)
Estriol	0.018095875	1.312021675	up	Steroid hormone biosynthesis(ko00140)
Levanbiose	0.007737725	1.355110906	up	Starch and sucrose metabolism(ko00500)
Galactocerebroside	0.024577983	1.187926697	up	Sphingolipid metabolism(ko00600)

FAD	0.007814947	1.333224175	up	Riboflavin metabolism(ko00740);;Vitamin digestion and absorption(ko04977)
5-Amino-6-(1-D-ribitylamino)uracil	0.005310012	1.402045755	up	Riboflavin metabolism(ko00740)
Angiotensin (5-7)	0.00014622	1.667850377	up	Renin-angiotensin system(ko04614)
Angiotensin III	0.018286484	1.244779243	up	Renin-angiotensin system(ko04614)
GTP	0.031313935	1.122198204	up	Rap1 signaling pathway(ko04015);;Sulfur relay system(ko04122);;Endocytosis(ko04144);;Riboflavin metabolism(ko00740);;Folate biosynthesis(ko00790);;Purine metabolism(ko00230);;Autophagy - animal(ko04140);;Ras signaling pathway(ko04014)
Orotidine 5'-phosphate	0.006774539	1.34408648	up	Pyrimidine metabolism(ko00240)
Xanthine	0.0044	1.3738	up	Purine metabolism(ko00230);;Caffeine metabolism(ko00232)

	03251	4254		
Xanthosine	0.011733842	1.255945276	up	Purine metabolism(ko00230);;ABC transporters(ko02010);;Caffeine metabolism(ko00232)
Inosine	0.014475587	1.245780168	up	Purine metabolism(ko00230);;ABC transporters(ko02010)
Hydroxymethylbilane	0.027352117	1.164982382	up	Porphyrin metabolism(ko00860)
Protochlorophyllide	0.047955876	1.114349832	up	Porphyrin metabolism(ko00860)
Biliverdin	0.019857689	1.246065135	up	Porphyrin metabolism(ko00860)
3-Hydroxyethylbacteriochlorophyllide a	0.011748579	1.318119103	up	Porphyrin metabolism(ko00860)

Cobinamide	0.00141212	1.509154218	up	Porphyrin metabolism(ko00860)
Hydrogenobyrinate a,c diamide	0.005384802	1.503611273	up	Porphyrin metabolism(ko00860)
Indoleglycerol phosphate	0.009063992	1.357340863	up	Phenylalanine, tyrosine and tryptophan biosynthesis(ko00400)
6-Deoxy-5-ketofructose 1-phosphate	0.028255376	1.180345011	up	Phenylalanine, tyrosine and tryptophan biosynthesis(ko00400)
Phenylacetylglutamine	0.002245104	1.596979601	up	Phenylalanine metabolism(ko00360)
Pantothenate	0.009446816	1.344375851	up	Pantothenate and CoA biosynthesis(ko00770);;Vitamin digestion and absorption(ko04977);;beta-Alanine metabolism(ko00410)
Pantetheine 4'-phosphate	0.0017	1.4861	up	Pantothenate and CoA biosynthesis(ko00770)

	23663	77152		
Dephospho-CoA	0.007096347	1.375664085	up	Pantothenate and CoA biosynthesis(ko00770)
D-Pantetheine 4'-phosphate	0.032007864	1.168615333	up	Pantothenate and CoA biosynthesis(ko00770)
Folinic acid	0.032066632	1.260820715	up	One carbon pool by folate(ko00670)
7,8-Dihydro-7-hydroxy-8-S-glutathionyl-benzo[a]pyrene	0.025916425	1.183819864	up	Metabolism of xenobiotics by cytochrome P450(ko00980);;Chemical carcinogenesis - DNA adducts(ko05204)
4-(Methylnitrosamino)-1-(1-oxido-3-pyridinyl)-1-butanone	0.03853303	1.125638416	up	Metabolism of xenobiotics by cytochrome P450(ko00980);;Chemical carcinogenesis - DNA adducts(ko05204)
(1R)-Hydroxy-(2R)-glutathionyl-1,2-dihydronaphthalene	0.004004732	1.378486495	up	Metabolism of xenobiotics by cytochrome P450(ko00980)

1-Nitro-5-glutathionyl-6-hydroxy-5,6-dihydronaphthalene	0.018897174	1.272463483	up	Metabolism of xenobiotics by cytochrome P450(ko00980)
5-Aminopentanal	0.001763568	1.522773847	up	Lysine degradation(ko00310)
6-Acetamido-2-oxohexanoate	0.000255744	1.602909414	up	Lysine degradation(ko00310)
D-Lysopine	0.015446424	1.324060461	up	Lysine degradation(ko00310)
Pyruvic acid	0.021871529	1.177006483	up	Insulin resistance(ko04931);;Cysteine and methionine metabolism(ko00270);;C5-Branched dibasic acid metabolism(ko00660);;Alanine, aspartate and glutamate metabolism(ko00250);;Phosphonate and phosphinate metabolism(ko00440);;Taurine and hypotaurine metabolism(ko00430);;Pyruvate metabolism(ko00620);;Pantothenate and CoA biosynthesis(ko00770);;Butanoate metabolism(ko00650);;Pentose and glucuronate interconversions(ko00040);;Glucagon signaling pathway(ko04922);;Glyoxylate and dicarboxylate metabolism(ko00630);;Thiamine metabolism(ko00730);;D-Amino acid metabolism(ko00470);;HIF-1 signaling pathway(ko04066);;Diabetic cardiomyopathy(ko05415);;Nicotinate and nicotinamide metabolism(ko00760);;Type II

				diabetes mellitus(ko04930);;Glycine, serine and threonine metabolism(ko00260);;Glycolysis / Gluconeogenesis(ko00010);;Phenylalanine metabolism(ko00360);;Valine, leucine and isoleucine biosynthesis(ko00290);;Tyrosine metabolism(ko00350);;Insulin secretion(ko04911);;Central carbon metabolism in cancer(ko05230);;Terpenoid backbone biosynthesis(ko00900);;Monobactam biosynthesis(ko00261);;Ascorbate and aldarate metabolism(ko00053);;AMPK signaling pathway(ko04152);;Pentose phosphate pathway(ko00030);;Arginine and proline metabolism(ko00330);;Citrate cycle (TCA cycle)(ko00020)
1-(5-Phosphoribosyl)imidazole-4-acetate	0.026862662	1.255799707	up	Histidine metabolism(ko00340)
4-(beta-Acetylaminoethyl)imidazole	0.040648493	1.197159623	up	Histidine metabolism(ko00340)
Arbutin	0.016293652	1.269515202	up	Glycolysis / Gluconeogenesis(ko00010)
Trypanothione disulfide	0.01300398	1.291090606	up	Glutathione metabolism(ko00480)
Trypanothione	0.0	1.3	up	Glutathione metabolism(ko00480)

	062<br>552<br>01	448<br>064<br>45		
Stachyose	0.0<br>243<br>951<br>74	1.2<br>564<br>573<br>86	up	Galactose metabolism(ko00052)
L-Fucose 1-phosphate	0.0<br>031<br>732<br>69	1.5<br>727<br>601<br>04	up	Fructose and mannose metabolism(ko00051);;Amino sugar and nucleotide sugar metabolism(ko00520)
L-Rhamnulose	0.0<br>010<br>652<br>08	1.5<br>544<br>194<br>91	up	Fructose and mannose metabolism(ko00051)
trans-Tetradec-2-enoyl-CoA	0.0<br>065<br>180<br>8	1.4<br>243<br>082<br>23	up	Fatty acid elongation(ko00062);;Fatty acid degradation(ko00071)
2-trans-Dodecenoyl-CoA	0.0<br>191<br>840<br>39	1.1<br>864<br>270<br>41	up	Fatty acid elongation(ko00062);;Fatty acid degradation(ko00071)
5'-Deoxy-5-fluorocytidine	0.0<br>007<br>415	1.6<br>281<br>360	up	Drug metabolism - other enzymes(ko00983)

	7	51		
floxuridine	0.022028246	1.190146503	up	Drug metabolism - other enzymes(ko00983)
Isonicotinoyl-NAD adduct	0.004964835	1.392701505	up	Drug metabolism - other enzymes(ko00983)
SN-38	0.018794152	1.289739833	up	Drug metabolism - other enzymes(ko00983)
NADP+	0.001932896	1.521565297	up	Diabetic cardiomyopathy(ko05415);;Nicotinate and nicotinamide metabolism(ko00760);;Alcoholic liver disease(ko04936);;Drug metabolism - other enzymes(ko00983);;Chemical carcinogenesis - reactive oxygen species(ko05208);;Aldosterone synthesis and secretion(ko04925);;Glutathione metabolism(ko00480);;Thyroid hormone synthesis(ko04918)
Linatine	0.019465501	1.29452308	up	D-Amino acid metabolism(ko00470)
S-Adenosylhomocysteine	0.023780109	1.254343503	up	Cysteine and methionine metabolism(ko00270);;Chemical carcinogenesis - reactive oxygen species(ko05208)

S-Adenosylmethioninamine	0.000940595	1.503087169	up	Cysteine and methionine metabolism(ko00270);;Arginine and proline metabolism(ko00330)
5'-Methylthioadenosine	9.55E-05	1.609511728	up	Cysteine and methionine metabolism(ko00270)
16-Oxopalmitate	0.031852734	1.240261224	up	Cutin, suberine and wax biosynthesis(ko00073)
Angiotensin (1-7)	0.004063671	1.418093594	up	Coronavirus disease - COVID-19(ko05171);;Renin-angiotensin system(ko04614)
Bradykinin	0.002563712	1.423450575	up	Complement and coagulation cascades(ko04610)
Glycocholate	0.013409862	1.262250636	up	Cholesterol metabolism(ko04979);;Primary bile acid biosynthesis(ko00120);;Bile secretion(ko04976)
Benzene	0.0211	1.2343	up	Chemical carcinogenesis - reactive oxygen species(ko05208)

	25828	80381		
7-Hydroxymethyl-12-methylbenz[a]anthracene	0.002159246	1.490247244	up	Chemical carcinogenesis - DNA adducts(ko05204);;Metabolism of xenobiotics by cytochrome P450(ko00980)
L-Proline	0.002780714	1.431943168	up	Central carbon metabolism in cancer(ko05230);;Aminoacyl-tRNA biosynthesis(ko00970);;Arginine and proline metabolism(ko00330);;ABC transporters(ko02010);;Protein digestion and absorption(ko04974);;D-Amino acid metabolism(ko00470);;Mineral absorption(ko04978)
3,6,8-Trimethylallantoin	0.00556272	1.372060837	up	Caffeine metabolism(ko00232)
2-Hydroxyparaconate	0.001577839	1.506759983	up	C5-Branched dibasic acid metabolism(ko00660)
Docosenoyl-CoA	0.020127375	1.333455923	up	Biosynthesis of unsaturated fatty acids(ko01040)
Montanoyl-CoA	0.024789589	1.309343649	up	Biosynthesis of unsaturated fatty acids(ko01040)

Docosanoyl-CoA	0.023587507	1.300295416	up	Biosynthesis of unsaturated fatty acids(ko01040)
(13Z,16Z)-Docosadienoic acid	0.002552622	1.476943035	up	Biosynthesis of unsaturated fatty acids(ko01040)
Levofloxacin	0.021030019	1.301402572	up	Bile secretion(ko04976)
BQ 123	9.50E-05	1.667670632	up	Bile secretion(ko04976)
Zalcitabine	0.020117537	1.348810257	up	Bile secretion(ko04976)
Tetracycline	0.016043242	1.254520897	up	Bile secretion(ko04976)
Vinblastine	0.0086	1.3093	up	Bile secretion(ko04976)

	30601	77207		
Digoxin	0.000301866	1.633037913	up	Bile secretion(ko04976)
Pantothenic acid	0.006337677	1.462919017	up	beta-Alanine metabolism(ko00410);;Vitamin digestion and absorption(ko04977);;Pantothenate and CoA biosynthesis(ko00770)
Serotonin	0.010308281	1.279606688	up	Axon regeneration(ko04361);;Tryptophan metabolism(ko00380);;Serotonergic synapse(ko04726);;Gap junction(ko04540);;Bile secretion(ko04976);;Taste transduction(ko04742);;Neuroactive ligand-receptor interaction(ko04080);;Inflammatory mediator regulation of TRP channels(ko04750);;Synaptic vesicle cycle(ko04721);;Chemical carcinogenesis - receptor activation(ko05207);;cAMP signaling pathway(ko04024)
L-Ascorbic acid	0.035996569	1.099187531	up	Ascorbate and aldarate metabolism(ko00053);;Glutathione metabolism(ko00480);;Vitamin digestion and absorption(ko04977);;HIF-1 signaling pathway(ko04066)
Ascorbic acid	0.003991387	1.39113758	up	Ascorbate and aldarate metabolism(ko00053);;Glutathione metabolism(ko00480);;Vitamin digestion and absorption(ko04977);;HIF-1 signaling pathway(ko04066)
D-Octopine	0.0042	1.4052	up	Arginine and proline metabolism(ko00330);;ABC transporters(ko02010)

	024<br>91	846<br>96		
CMP-pseudaminic acid	0.0<br>297<br>342<br>97	1.2<br>120<br>421<br>96	up	Amino sugar and nucleotide sugar metabolism(ko00520)
Undecaprenyl phosphate alpha-L-Ara4N	0.0<br>208<br>035<br>59	1.2<br>621<br>580<br>42	up	Amino sugar and nucleotide sugar metabolism(ko00520)
Pseudaminic acid	0.0<br>315<br>890<br>62	1.2<br>608<br>254<br>68	up	Amino sugar and nucleotide sugar metabolism(ko00520)
OPC8-CoA	0.0<br>321<br>959<br>75	1.2<br>510<br>980<br>78	up	alpha-Linolenic acid metabolism(ko00592)
D-Glucosamine 6-phosphate	0.0<br>091<br>884<br>29	1.4<br>270<br>595<br>94	up	Alanine, aspartate and glutamate metabolism(ko00250);;Amino sugar and nucleotide sugar metabolism(ko00520);;Diabetic cardiomyopathy(ko05415);;Insulin resistance(ko04931)
L-Isoleucine	0.0<br>017<br>403<br>69	1.4<br>608<br>439<br>73	up	ABC transporters(ko02010);;Valine, leucine and isoleucine biosynthesis(ko00290);;Mineral absorption(ko04978);;Valine, leucine and isoleucine degradation(ko00280);;Shigellosis(ko05131);;Central carbon metabolism in cancer(ko05230);;Protein digestion and absorption(ko04974);;Aminoacyl-tRNA

				biosynthesis(ko00970)
4-Amino-5-hydroxymethyl-2-methylpyrimidine	0.038490975	1.108968741	up	ABC transporters(ko02010);;Thiamine metabolism(ko00730)
Cytidine	0.003203205235	1.432153919	up	ABC transporters(ko02010);;Pyrimidine metabolism(ko00240)
Maltotriose	0.01614197	1.367882667	up	ABC transporters(ko02010);;Carbohydrate digestion and absorption(ko04973)
Nopaline	0.00140875 6	1.49125226	up	ABC transporters(ko02010);;Arginine and proline metabolism(ko00330)
Isomaltotriose	0.02413296	1.245279171	up	ABC transporters(ko02010)
alpha-1,5-L-Arabinotriose	0.027919473	1.224943937	up	ABC transporters(ko02010)
name	Pva	VIP	re	KEGG_pathway_annotation

	lue		gulated	
Phylloquinone	0.049707458	1.09106684	down	Vitamin digestion and absorption(ko04977);;Ubiquinone and other terpenoid-quinone biosynthesis(ko00130)
Vitamin K1	0.002497711	1.484253127	down	Vitamin digestion and absorption(ko04977);;Ubiquinone and other terpenoid-quinone biosynthesis(ko00130)
dl-alpha-Tocopherol nicotinate	0.033846313	1.139803851	down	Vitamin digestion and absorption(ko04977)
4-Hydroxy-L-threonine	0.000952083	1.581552608	down	Vitamin B6 metabolism(ko00750)
(R)-3-Amino-2-methylpropanoate	0.032459666	1.205445586	down	Valine, leucine and isoleucine degradation(ko00280);;Pyrimidine metabolism(ko00240)
3-Hydroxyisovalerate	0.0008	1.5156	down	Valine, leucine and isoleucine degradation(ko00280)

	78977	62788		
alpha-Isopropylmalate	0.025371715	1.185745344	down	Valine, leucine and isoleucine biosynthesis(ko00290);;Pyruvate metabolism(ko00620)
alpha-Tocopherol	0.033976584	1.183653104	down	Ubiquinone and other terpenoid-quinone biosynthesis(ko00130);;Vitamin digestion and absorption(ko04977);;Ferroptosis(ko04216)
Dihydroshikonofuran	0.001037033	1.605276657	down	Ubiquinone and other terpenoid-quinone biosynthesis(ko00130)
2-Phytyl-1,4-naphthoquinone	0.006401911	1.501869009	down	Ubiquinone and other terpenoid-quinone biosynthesis(ko00130)
beta-Tocotrienol	0.040957852	1.138503492	down	Ubiquinone and other terpenoid-quinone biosynthesis(ko00130)
Gentisate aldehyde	0.041376008	1.066407168	down	Tyrosine metabolism(ko00350)

gamma-Oxalocrotonate	0.018430414	1.222926172	down	Tryptophan metabolism(ko00380)
2-Aminomuconic acid	0.025361411	1.224783505	down	Tryptophan metabolism(ko00380)
4,8-Dihydroxyquinoline	0.000826556	1.511681261	down	Tryptophan metabolism(ko00380)
Oxoglutaric acid	0.001301058	1.480848141	down	Taurine and hypotaurine metabolism(ko00430);;GABAergic synapse(ko04727);;C5-Branched dibasic acid metabolism(ko00660);;Proximal tubule bicarbonate reclamation(ko04964);;Alanine, aspartate and glutamate metabolism(ko00250);;Glucagon signaling pathway(ko04922);;Pentose and glucuronate interconversions(ko00040);;Bile secretion(ko04976);;Butanoate metabolism(ko00650);;D-Amino acid metabolism(ko00470);;HIF-1 signaling pathway(ko04066);;Ascorbate and aldarate metabolism(ko00053);;Glyoxylate and dicarboxylate metabolism(ko00630);;Arginine biosynthesis(ko00220);;Central carbon metabolism in cancer(ko05230);;Histidine metabolism(ko00340);;Citrate cycle (TCA cycle)(ko00020);;Lysine degradation(ko00310);;Lysine biosynthesis(ko00300)
L-Malic acid	0.0375425	1.1357690	down	Taste transduction(ko04742);;Glucagon signaling pathway(ko04922);;Citrate cycle (TCA cycle)(ko00020);;Pathways in cancer(ko05200);;Renal cell carcinoma(ko05211);;Pyruvate metabolism(ko00620);;Central carbon metabolism in

	96	37		cancer(ko05230);;Proximal tubule bicarbonate reclamation(ko04964);;Glyoxylate and dicarboxylate metabolism(ko00630)
L-Homocysteine	0.031513514	1.181139086	down	Sulfur metabolism(ko00920);;Cysteine and methionine metabolism(ko00270);;NOD-like receptor signaling pathway(ko04621);;Antifolate resistance(ko01523)
O-Succinyl-L-homoserine	0.005435108	1.480788169	down	Sulfur metabolism(ko00920);;Cysteine and methionine metabolism(ko00270)
7alpha-Hydroxydehydroepiandrosterone	0.034705448	1.127284886	down	Steroid hormone biosynthesis(ko00140)
Cholesterol sulfate	0.028784385	1.107737881	down	Steroid hormone biosynthesis(ko00140)
22(R)-Hydroxycholesterol	0.04874375	1.027466964	down	Steroid hormone biosynthesis(ko00140)
3alpha,11beta,21-Trihydroxy-20-oxo-5beta-pregnan-18-al	0.040941401	1.195744005	down	Steroid hormone biosynthesis(ko00140)

Cortolone	0.0139610 1	1.3896068 47	down	Steroid hormone biosynthesis(ko00140)
Androsterone	0.0058970 31	1.3856280 62	down	Steroid hormone biosynthesis(ko00140)
11-Dehydrocorticosterone	0.0130925 18	1.3522597 72	down	Steroid hormone biosynthesis(ko00140)
4-Methylpentanal	0.0325514 56	1.1958888 68	down	Steroid hormone biosynthesis(ko00140)
Pregnanediol	0.0079441 9	1.3324927 81	down	Steroid hormone biosynthesis(ko00140)
3beta-Hydroxypregn-5-en-20-one sulfate	0.0058415 31	1.4499282 51	down	Steroid hormone biosynthesis(ko00140)
Calcidiol	0.0035	1.4225	down	Steroid biosynthesis(ko00100);;Tuberculosis(ko05152);;Parathyroid hormone synthesis, secretion and action(ko04928)

	2098	56071		
Calcitetrol	0.044897044	1.147868169	down	Steroid biosynthesis(ko00100);;Parathyroid hormone synthesis, secretion and action(ko04928)
7-dehydrocholesterol	0.012626818	1.303032955	down	Steroid biosynthesis(ko00100);;Insect hormone biosynthesis(ko00981)
Ergosta-5,7,22,24(28)-tetraen-3beta-ol	0.017251744	1.19505335	down	Steroid biosynthesis(ko00100)
24-Ethylidenelophenol	0.011925892	1.323682331	down	Steroid biosynthesis(ko00100)
Ergosterol	0.000911671	1.545796844	down	Steroid biosynthesis(ko00100)
5-Dehydroepisterol	0.001790419	1.488787013	down	Steroid biosynthesis(ko00100)

4alpha-Methylzymosterol	0.0400349 23	1.0915767 5	down	Steroid biosynthesis(ko00100)
4alpha-Methyl-5alpha-ergosta-8,14,24(28)-trien-3beta-ol	0.0029195 15	1.4247514 63	down	Steroid biosynthesis(ko00100)
Campesterol	0.0060076 15	1.4348901 82	down	Steroid biosynthesis(ko00100)
Stigmasterol	0.0199602 96	1.2139642 61	down	Steroid biosynthesis(ko00100)
4alpha-carboxy-4beta-methyl-5alpha-cholesta-8,24-dien-3beta-ol	0.0103071 17	1.4148339 95	down	Steroid biosynthesis(ko00100)
3-Ketosucrose	0.0007121 75	1.5239619 69	down	Starch and sucrose metabolism(ko00500)
5-Amino-6-(5'-phosphoribosylamino)uracil	0.0213	1.2596	down	Riboflavin metabolism(ko00740)

	7037	18489		
all-trans-3,4-Didehydroretinol	0.001408861	1.597406284	down	Retinol metabolism(ko00830)
ADP	0.021044131	1.264741758	down	Renin secretion(ko04924);;Taste transduction(ko04742);;Purine metabolism(ko00230);;Lysosome(ko04142);;AMPK signaling pathway(ko04152);;Thermogenesis(ko04714);;Oxidative phosphorylation(ko00190);;FoxO signaling pathway(ko04068);;Parkinson disease(ko05012);;Pathways of neurodegeneration - multiple diseases(ko05022);;Neuroactive ligand-receptor interaction(ko04080);;Platelet activation(ko04611);;Amyotrophic lateral sclerosis(ko05014)
(R)-S-Lactoylglutathione	0.027963128	1.140509503	down	Pyruvate metabolism(ko00620)
2'-Deoxy-5-hydroxymethylcytidine-5'-triphosphate	0.011421734	1.29835647	down	Pyrimidine metabolism(ko00240)
dCDP	0.005016438	1.374818565	down	Pyrimidine metabolism(ko00240)
CMP	0.0	1.1	do	Pyrimidine metabolism(ko00240)

	35876334	18930558	wn	
Thymidine	0.040500232	1.094709178	down	Pyrimidine metabolism(ko00240)
(Z)-3-Ureidoacrylate peracid	0.003189365	1.402032691	down	Pyrimidine metabolism(ko00240)
5-Methylcytosine	0.001276644	1.476039047	down	Pyrimidine metabolism(ko00240)
(R)(-)-Allantoin	0.027120443	1.258135869	down	Purine metabolism(ko00230)
N-Formiminoglycine	0.004215008	1.435093631	down	Purine metabolism(ko00230)
dGDP	0.0019213	1.4922975	down	Purine metabolism(ko00230)

	66	83		
dGMP	0.00222678	1.509956458	down	Purine metabolism(ko00230)
Deoxyadenosine monophosphate	0.00675122	1.447317049	down	Purine metabolism(ko00230)
1-(5-Phospho-D-ribosyl)-5-amino-4-imidazolecarboxylate	0.000269249	1.708402451	down	Purine metabolism(ko00230)
5-Amino-4-imidazole carboxylate	0.020723037	1.22400528	down	Purine metabolism(ko00230)
Butyric acid	0.024377447	1.187291534	down	Protein digestion and absorption(ko04974);;Carbohydrate digestion and absorption(ko04973);;Butanoate metabolism(ko00650)
p-cresol	6.87E-05	1.655483708	down	Protein digestion and absorption(ko04974)
Androstenedione	0.0	1.4	do	Prostate cancer(ko05215);;Ovarian steroidogenesis(ko04913);;Endocrine

	05152663	44414653	wn	resistance(ko01522);;Steroid hormone biosynthesis(ko00140);;Prolactin signaling pathway(ko04917);;Pathways in cancer(ko05200)
3alpha,7alpha,12alpha,26-Tetrahydroxy-5beta-cholestane	0.002272358	1.466884957	down	Primary bile acid biosynthesis(ko00120)
4-Cholesten-7alpha,12alpha-diol-3-one	0.034174569	1.155986488	down	Primary bile acid biosynthesis(ko00120)
7alpha-Hydroxy-3-oxo-4-cholestenoate	4.03E-05	1.756644752	down	Primary bile acid biosynthesis(ko00120)
3alpha,7alpha-Dihydroxy-5beta-cholestanate	0.012603831	1.380562979	down	Primary bile acid biosynthesis(ko00120)
3alpha,7alpha,12alpha-Trihydroxy-5beta-cholestane	0.008578304	1.361720238	down	Primary bile acid biosynthesis(ko00120)
7alpha,12alpha-Dihydroxy-5beta-cholestan-3-one	0.0383443	1.0914610	down	Primary bile acid biosynthesis(ko00120)

	4	61		
8(S)-HETE	0.0011670 15	1.488309425	down	PPAR signaling pathway(ko03320);;Arachidonic acid metabolism(ko00590)
Biliverdin-IX-delta	0.049311681	1.204519105	down	Porphyrin metabolism(ko00860)
3-Hydroxyethylchlorophyllide a	0.009928014	1.301105557	down	Porphyrin metabolism(ko00860)
5-Oxo-delta-bilirubin	0.000998471	1.554039678	down	Porphyrin metabolism(ko00860)
Biliverdin-IX-beta	0.016262234	1.404106731	down	Porphyrin metabolism(ko00860)
N-Acetylbialaphos	0.011632011	1.397231076	down	Phosphonate and phosphinate metabolism(ko00440)
Demethylphosphinothricin	0.0	1.2	do	Phosphonate and phosphinate metabolism(ko00440)

	140<br>779<br>17	967<br>350<br>41	wn	
Chorismate	0.0<br>001<br>478<br>37	1.6<br>981<br>903<br>4	do<br>wn	Phenylalanine, tyrosine and tryptophan biosynthesis(ko00400);;Ubiquinone and other terpenoid-quinone biosynthesis(ko00130);;Folate biosynthesis(ko00790)
L-quinate	0.0<br>016<br>272<br>74	1.4<br>669<br>131<br>69	do<br>wn	Phenylalanine, tyrosine and tryptophan biosynthesis(ko00400)
Phenylacetaldehyde	0.0<br>017<br>011<br>11	1.4<br>602<br>742<br>63	do<br>wn	Phenylalanine metabolism(ko00360)
6-Aminopenicillanate	0.0<br>397<br>724<br>65	1.1<br>112<br>119<br>42	do<br>wn	Penicillin and cephalosporin biosynthesis(ko00311)
Phosphoric acid	0.0<br>106<br>630<br>16	1.2<br>838<br>383<br>8	do<br>wn	Pathways of neurodegeneration - multiple diseases(ko05022);;Parkinson disease(ko05012);;Oxidative phosphorylation(ko00190);;Mineral absorption(ko04978);;ABC transporters(ko02010);;Parathyroid hormone synthesis, secretion and action(ko04928)
Pyrophosphate	0.0<br>138<br>471	1.2<br>564<br>908	do<br>wn	Pathways of neurodegeneration - multiple diseases(ko05022);;Oxidative phosphorylation(ko00190);;Parkinson disease(ko05012)

	82	07		
Dopamine quinone	0.001002187	1.53342077	down	Parkinson disease(ko05012)
(R)-4-Dehydropantoate	0.008372964	1.3304301	down	Pantothenate and CoA biosynthesis(ko00770)
Cyclic ADP-ribose	0.036777149	1.112424405	down	Pancreatic secretion(ko04972);;Calcium signaling pathway(ko04020);;Oxytocin signaling pathway(ko04921);;Salivary secretion(ko04970)
Carbamoyl phosphate	0.00172085	1.451775106	down	Nitrogen metabolism(ko00910);;Purine metabolism(ko00230);;Arginine biosynthesis(ko00220);;Pyrimidine metabolism(ko00240);;Alanine, aspartate and glutamate metabolism(ko00250)
Nicotine	0.01888669	1.249090401	down	Nicotine addiction(ko05033);;Thermogenesis(ko04714);;Metabolism of xenobiotics by cytochrome P450(ko00980);;Chemical carcinogenesis - receptor activation(ko05207)
5-(N-Methyl-4,5-dihydro-1H-pyrrol-2-yl)pyridin-2-ol	0.038975846	1.234773746	down	Nicotinate and nicotinamide metabolism(ko00760)
1-Nitro-7-glutathionyl-8-hydro	0.0	1.6	do	Metabolism of xenobiotics by cytochrome P450(ko00980)

xy-7,8-dihydronaphthalene	00199954	07152002	wn	
S-[2-(N7-Guanyl)ethyl]-N-acetyl-L-cysteine	0.015940035	1.255035066	down	Metabolism of xenobiotics by cytochrome P450(ko00980)
Bromobenzene-2,3-oxide	0.041892331	1.106708992	down	Metabolism of xenobiotics by cytochrome P450(ko00980)
6-[2,3-Dihydroxy-1-(hydroxymethyl)propyl]-1,2-dihydro-7-hydroxy-9-methoxy-cyclopenta[c][1]benzopyran-3,4-dione	0.00504022	1.446710878	down	Metabolism of xenobiotics by cytochrome P450(ko00980)
N6,N6,N6-Trimethyl-L-lysine	0.005512086	1.400190263	down	Lysine degradation(ko00310)
L-Pipecolate	0.002301606	1.440179712	down	Lysine degradation(ko00310)
(2R,3R)-3-Methylornithinyl-N6-lysine	0.0038101	1.4183014	down	Lysine biosynthesis(ko00300)

	79	47		
(Z)-But-1-ene-1,2,4-tricarboxylate	6.28E-05	1.741015076	down	Lysine biosynthesis(ko00300)
Sirolimus	0.032968915	1.118117543	down	Longevity regulating pathway - worm(ko04212);;Cellular senescence(ko04218);;Longevity regulating pathway(ko04211)
Delta4-Dafachronic acid	0.03124883	1.146888933	down	Longevity regulating pathway - worm(ko04212)
Dihydrolipoate	0.003029255	1.584324602	down	Lipoic acid metabolism(ko00785)
13(S)-HODE	0.040672782	1.077335789	down	Linoleic acid metabolism(ko00591);;PPAR signaling pathway(ko03320)
Dihomo-gamma-linolenate	0.003362399	1.369316546	down	Linoleic acid metabolism(ko00591);;Biosynthesis of unsaturated fatty acids(ko01040)
9,10,13-TriHOME	0.0	1.4	do	Linoleic acid metabolism(ko00591)

	05652245	32192322	wn	
9,12,13-TriHOME	0.00932461	1.301812704	down	Linoleic acid metabolism(ko00591)
9,10-Epoxy-13-hydroxy-11-octadecenoate	0.002179933	1.449649804	down	Linoleic acid metabolism(ko00591)
(+)-Camphor	0.019582851	1.252800467	down	Inflammatory mediator regulation of TRP channels(ko04750)
Anserine	0.004688921	1.486719966	down	Histidine metabolism(ko00340);;beta-Alanine metabolism(ko00410)
Dihydrourocanate	0.008444473	1.321657406	down	Histidine metabolism(ko00340)
L-Histidinol	0.0018221	1.4733823	down	Histidine metabolism(ko00340)

	32	55		
4-Hydroxynonenal	0.005459699	1.347889679	down	Hepatocellular carcinoma(ko05225);;Pathways in cancer(ko05200)
Citric acid	0.017682652	1.282008741	down	Glyoxylate and dicarboxylate metabolism(ko00630);;Alanine, aspartate and glutamate metabolism(ko00250);;Central carbon metabolism in cancer(ko05230);;Glucagon signaling pathway(ko04922);;Citrate cycle (TCA cycle)(ko00020);;Taste transduction(ko04742)
4-Hydroxy-2-oxoglutaric acid	0.011569045	1.353334404	down	Glyoxylate and dicarboxylate metabolism(ko00630)
Pyroglutamic acid	0.008696762	1.300389938	down	Glutathione metabolism(ko00480)
Glutathionylspermine	0.011742987	1.435826251	down	Glutathione metabolism(ko00480)
L-Fucono-1,5-lactone	0.000525155	1.575961052	down	Fructose and mannose metabolism(ko00051)
Sepiapterin	0.0	1.5	do	Folate biosynthesis(ko00790)

	01520765	49709112	wn	
Neopterin	0.00206874	1.447668949	down	Folate biosynthesis(ko00790)
Tetrahydrobiopterin	0.034069866	1.176794754	down	Fluid shear stress and atherosclerosis(ko05418);;Folate biosynthesis(ko00790)
1-Octadecanoyl-2-(7Z,10Z,13Z,16Z-docosatetraenoyl)-sn-glycero-3-phosphoethanolamine	0.026938735	1.270843166	down	Ferroptosis(ko04216)
1-Octadecanoyl-sn-glycero-3-phosphoethanolamine	0.021918897	1.203192356	down	Ferroptosis(ko04216)
trans,cis-Lauro-2,6-dienoyl-CoA	0.000120337	1.639748148	down	Fatty acid degradation(ko00071)
Decanoic acid	0.0287605	1.1470777	down	Fatty acid biosynthesis(ko00061)

	39	76		
(9Z)-Hexadecenoic acid	0.04967337	1.030687612	down	Fatty acid biosynthesis(ko00061)
5-Fluorodeoxyuridine	0.046660467	1.163808551	down	Drug metabolism - other enzymes(ko00983)
alpha-Fluoro-beta-ureidopropionic acid	0.008241891	1.342966469	down	Drug metabolism - other enzymes(ko00983)
Fluoroacetic acid	0.005278706	1.425022844	down	Drug metabolism - other enzymes(ko00983)
Carmofur	0.003005594	1.487628494	down	Drug metabolism - other enzymes(ko00983)
Endoxifen	0.019641446	1.327283765	down	Drug metabolism - cytochrome P450(ko00982);;Endocrine resistance(ko01522)
S-Adenosyl-L-homocysteine	0.0	1.4	do	Cysteine and methionine metabolism(ko00270);;Chemical carcinogenesis - reactive

	016 133 24	712 449 37	wn	oxygen species(ko05208)
Behenic acid	0.0 298 327 39	1.1 544 695 86	do wn	Cutin, suberine and wax biosynthesis(ko00073);;Biosynthesis of unsaturated fatty acids(ko01040)
22-Hydroxydocosanoic acid	0.0 039 467 44	1.4 229 715 29	do wn	Cutin, suberine and wax biosynthesis(ko00073)
17alpha-Hydroxypregnenolone	0.0 163 396 47	1.2 315 682 64	do wn	Cushing syndrome(ko04934);;Steroid hormone biosynthesis(ko00140);;Cortisol synthesis and secretion(ko04927);;Ovarian steroidogenesis(ko04913)
Cholesterol	0.0 018 356 72	1.4 791 452 93	do wn	Cushing syndrome(ko04934);;Lipid and atherosclerosis(ko05417);;Pathways in cancer(ko05200);;Cholesterol metabolism(ko04979);;Steroid biosynthesis(ko00100);;Steroid hormone biosynthesis(ko00140);;Bile secretion(ko04976);;Primary bile acid biosynthesis(ko00120);;Ovarian steroidogenesis(ko04913);;Cortisol synthesis and secretion(ko04927);;Insect hormone biosynthesis(ko00981);;Basal cell carcinoma(ko05217);;Aldosterone synthesis and secretion(ko04925);;Vitamin digestion and absorption(ko04977);;Fat digestion and absorption(ko04975)
Pregnenolone	0.0 024 769	1.5 261 329	do wn	Cortisol synthesis and secretion(ko04927);;Ovarian steroidogenesis(ko04913);;Steroid hormone biosynthesis(ko00140);;Aldosterone synthesis and secretion(ko04925);;Cushing syndrome(ko04934)

	41	13		
Oxidized glutathione	0.026359621	1.251975285	down	Chemical carcinogenesis - reactive oxygen species(ko05208);;Glutathione metabolism(ko00480);;Ferroptosis(ko04216);;Thyroid hormone synthesis(ko04918);;Diabetic cardiomyopathy(ko05415)
L-Asparagine	0.027135317	1.151253534	down	Central carbon metabolism in cancer(ko05230);;Aminoacyl-tRNA biosynthesis(ko00970);;Alanine, aspartate and glutamate metabolism(ko00250);;Protein digestion and absorption(ko04974);;Mineral absorption(ko04978)
cis-Aconitic acid	0.027653347	1.24455503	down	C5-Branched dibasic acid metabolism(ko00660);;Glyoxylate and dicarboxylate metabolism(ko00630);;Citrate cycle (TCA cycle)(ko00020)
4-Hydroxybutanoic acid	0.002186151	1.456089837	down	Butanoate metabolism(ko00650)
KAPA	0.012869963	1.296539092	down	Biotin metabolism(ko00780)
8-Amino-7-oxononanoate	0.003660877	1.503059958	down	Biotin metabolism(ko00780)
7,8-Diaminononanoate	0.0	1.4	do	Biotin metabolism(ko00780)

	01964489	73377635	wn	
8-Amino-7-oxononanoic acid	0.004309825	1.441165983	down	Biotin metabolism(ko00780)
Adrenic acid	0.016562229	1.30095578	down	Biosynthesis of unsaturated fatty acids(ko01040);;Ferroptosis(ko04216)
(9Z)-Octadecenoic acid	0.002424027	1.484729424	down	Biosynthesis of unsaturated fatty acids(ko01040);;Cutin, suberine and wax biosynthesis(ko00073);;Fatty acid biosynthesis(ko00061);;Longevity regulating pathway - worm(ko04212)
Docosanoic acid	0.012954101	1.266474855	down	Biosynthesis of unsaturated fatty acids(ko01040);;Cutin, suberine and wax biosynthesis(ko00073)
Docosahexaenoic acid	0.020059262	1.249913277	down	Biosynthesis of unsaturated fatty acids(ko01040)
Tetracosanoic acid	0.0444758	1.1441714	down	Biosynthesis of unsaturated fatty acids(ko01040)

	59	59		
Spermine	0.012577666	1.2688678	down	Bile secretion(ko04976);;Arginine and proline metabolism(ko00330);;Pantothenate and CoA biosynthesis(ko00770);;beta-Alanine metabolism(ko00410);;Glutathione metabolism(ko00480)
Rifampicin	0.034050814	1.138359705	down	Bile secretion(ko04976)
L-Histidine	0.022919946	1.222376367	down	beta-Alanine metabolism(ko00410);;Histidine metabolism(ko00340);;Protein digestion and absorption(ko04974);;D-Amino acid metabolism(ko00470);;ABC transporters(ko02010);;Aminoacyl-tRNA biosynthesis(ko00970);;Central carbon metabolism in cancer(ko05230)
beta-Alanyl-L-arginine	0.022089453	1.275225809	down	beta-Alanine metabolism(ko00410)
Prostaglandin D2	4.02E-06	1.745509368	down	Asthma(ko05310);;Fc epsilon RI signaling pathway(ko04664);;Neuroactive ligand-receptor interaction(ko04080);;Serotonergic synapse(ko04726);;Arachidonic acid metabolism(ko00590);;African trypanosomiasis(ko05143)
L-Arabinono-1,4-lactone	0.01458613	1.260072848	down	Ascorbate and aldarate metabolism(ko00053)
Threonate	0.0	1.4	do	Ascorbate and aldarate metabolism(ko00053)

	11666376	43598118	wn	
Feruloylputrescine	0.007303058	1.326993994	down	Arginine and proline metabolism(ko00330)
L-4-Hydroxyglutamate semialdehyde	0.003082068	1.414024764	down	Arginine and proline metabolism(ko00330)
Prostaglandin G2	0.007717893	1.378137358	down	Arachidonic acid metabolism(ko00590);;Serotonergic synapse(ko04726);;Platelet activation(ko04611)
(15S)-15-Hydroxy-5,8,11-cis-13-trans-eicosatetraenoate	0.04051805	1.117874495	down	Arachidonic acid metabolism(ko00590);;Inflammatory mediator regulation of TRP channels(ko04750)
20-COOH-Leukotriene B4	0.045998707	1.150348008	down	Arachidonic acid metabolism(ko00590)
16(R)-HETE	0.0091244	1.3189621	down	Arachidonic acid metabolism(ko00590)

	52	85		
12-Keto-leukotriene B4	0.003323692	1.471928582	down	Arachidonic acid metabolism(ko00590)
Sphingosine 1-phosphate	0.003671735	1.567326769	down	Apelin signaling pathway(ko04371);;Neuroactive ligand-receptor interaction(ko04080);;Sphingolipid metabolism(ko00600);;Phospholipase D signaling pathway(ko04072);;Tuberculosis(ko05152);;Fc gamma R-mediated phagocytosis(ko04666);;Calcium signaling pathway(ko04020);;Sphingolipid signaling pathway(ko04071)
L-Pyrrolysine	0.011637258	1.29423527	down	Aminoacyl-tRNA biosynthesis(ko00970);;Lysine biosynthesis(ko00300);;Protein digestion and absorption(ko04974)
N-Acetylneuraminate	0.021556583	1.28164344	down	Amino sugar and nucleotide sugar metabolism(ko00520)
D-Glucosamine	0.00321334	1.596258508	down	Amino sugar and nucleotide sugar metabolism(ko00520)
2(R)-HOT	0.004399826	1.442443773	down	alpha-Linolenic acid metabolism(ko00592)

9-Hydroxy-12-oxo-15(Z)-octadecenoic acid	0.00333883	1.419673051	down	alpha-Linolenic acid metabolism(ko00592)
Methyl jasmonate	0.00013361	1.637150126	down	alpha-Linolenic acid metabolism(ko00592)
Isoproterenol	0.008333105	1.349589037	down	Adrenergic signaling in cardiomyocytes(ko04261)
Norfloxacin	0.009937143	1.333124934	down	ABC transporters(ko02010)
Retinol	0.022748475	1.270179771	up	Vitamin digestion and absorption(ko04977);;Retinol metabolism(ko00830)
Vitamin A	0.045755897	1.091223109	up	Vitamin digestion and absorption(ko04977);;Retinol metabolism(ko00830)
20-HETE	0.0038	1.5390	up	Vascular smooth muscle contraction(ko04270);;Arachidonic acid metabolism(ko00590)

	92129	43141		
Acetoacetate	0.013354144	1.393176145	up	Valine, leucine and isoleucine degradation(ko00280);;Lysine degradation(ko00310);;Tyrosine metabolism(ko00350);;Butanoate metabolism(ko00650)
trans-Cinnamic acid	0.002579295	1.420064955	up	Ubiquinone and other terpenoid-quinone biosynthesis(ko00130);;Phenylalanine metabolism(ko00360)
3''-Hydroxy-geranylhydroquinone	0.013136217	1.228746137	up	Ubiquinone and other terpenoid-quinone biosynthesis(ko00130)
alpha-Tocotrienol	0.017773305	1.205869269	up	Ubiquinone and other terpenoid-quinone biosynthesis(ko00130)
Phenol	0.026976334	1.157662638	up	Tyrosine metabolism(ko00350);;Chemical carcinogenesis - reactive oxygen species(ko05208);;Protein digestion and absorption(ko04974)
N-Acetyl-5-hydroxytryptamine	0.001927495	1.462945749	up	Tryptophan metabolism(ko00380)

Isopentenyl phosphate	0.0325674 96	1.1349248 75	up	Terpenoid backbone biosynthesis(ko00900)
Salicin	0.0013258 34	1.5246010 26	up	Taste transduction(ko04742);;Glycolysis / Gluconeogenesis(ko00010)
2-Methoxy-estradiol-17beta 3-glucuronide	0.0004475 53	1.6104604 96	up	Steroid hormone biosynthesis(ko00140)
Estriol	0.0180958 75	1.3120216 75	up	Steroid hormone biosynthesis(ko00140)
Levanbiose	0.0077377 25	1.3551109 06	up	Starch and sucrose metabolism(ko00500)
Galactocerebroside	0.0245779 83	1.1879266 97	up	Sphingolipid metabolism(ko00600)
FAD	0.0078	1.3332	up	Riboflavin metabolism(ko00740);;Vitamin digestion and absorption(ko04977)

	14947	24175		
5-Amino-6-(1-D-ribitylamino)uracil	0.005310012	1.402045755	up	Riboflavin metabolism(ko00740)
Angiotensin (5-7)	0.00014622	1.667850377	up	Renin-angiotensin system(ko04614)
Angiotensin III	0.018286484	1.244779243	up	Renin-angiotensin system(ko04614)
GTP	0.031313935	1.122198204	up	Rap1 signaling pathway(ko04015);;Sulfur relay system(ko04122);;Endocytosis(ko04144);;Riboflavin metabolism(ko00740);;Folate biosynthesis(ko00790);;Purine metabolism(ko00230);;Autophagy - animal(ko04140);;Ras signaling pathway(ko04014)
Orotidine 5'-phosphate	0.006774539	1.34408648	up	Pyrimidine metabolism(ko00240)
Xanthine	0.004403251	1.37384254	up	Purine metabolism(ko00230);;Caffeine metabolism(ko00232)

Xanthosine	0.0<br>117<br>338<br>42	1.2<br>559<br>452<br>76	up	Purine metabolism(ko00230);;ABC transporters(ko02010);;Caffeine metabolism(ko00232)
Inosine	0.0<br>144<br>755<br>87	1.2<br>457<br>801<br>68	up	Purine metabolism(ko00230);;ABC transporters(ko02010)
Hydroxymethylbilane	0.0<br>273<br>521<br>17	1.1<br>649<br>823<br>82	up	Porphyrin metabolism(ko00860)
Protochlorophyllide	0.0<br>479<br>558<br>76	1.1<br>143<br>498<br>32	up	Porphyrin metabolism(ko00860)
Biliverdin	0.0<br>198<br>576<br>89	1.2<br>460<br>651<br>35	up	Porphyrin metabolism(ko00860)
3-Hydroxyethylbacteriochlorophyllide a	0.0<br>117<br>485<br>79	1.3<br>181<br>191<br>03	up	Porphyrin metabolism(ko00860)
Cobinamide	0.0<br>014	1.5<br>091	up	Porphyrin metabolism(ko00860)

	1212	54218		
Hydrogenobyrinate a,c diamide	0.005384802	1.503611273	up	Porphyrin metabolism(ko00860)
Indoleglycerol phosphate	0.009063992	1.357340863	up	Phenylalanine, tyrosine and tryptophan biosynthesis(ko00400)
6-Deoxy-5-ketofructose 1-phosphate	0.028255376	1.180345011	up	Phenylalanine, tyrosine and tryptophan biosynthesis(ko00400)
Phenylacetylglutamine	0.002245104	1.596979601	up	Phenylalanine metabolism(ko00360)
Pantothenate	0.009446816	1.344375851	up	Pantothenate and CoA biosynthesis(ko00770);;Vitamin digestion and absorption(ko04977);;beta-Alanine metabolism(ko00410)
Pantetheine 4'-phosphate	0.001723663	1.486177152	up	Pantothenate and CoA biosynthesis(ko00770)

Dephospho-CoA	0.007096347	1.375664085	up	Pantothenate and CoA biosynthesis(ko00770)
D-Pantetheine 4'-phosphate	0.032007864	1.168615333	up	Pantothenate and CoA biosynthesis(ko00770)
Folinic acid	0.032066632	1.260820715	up	One carbon pool by folate(ko00670)
7,8-Dihydro-7-hydroxy-8-S-glutathionyl-benzo[a]pyrene	0.025916425	1.183819864	up	Metabolism of xenobiotics by cytochrome P450(ko00980);;Chemical carcinogenesis - DNA adducts(ko05204)
4-(Methylnitrosamino)-1-(1-oxido-3-pyridinyl)-1-butanone	0.03853303	1.125638416	up	Metabolism of xenobiotics by cytochrome P450(ko00980);;Chemical carcinogenesis - DNA adducts(ko05204)
(1R)-Hydroxy-(2R)-glutathionyl-1,2-dihydronaphthalene	0.004004732	1.378486495	up	Metabolism of xenobiotics by cytochrome P450(ko00980)
1-Nitro-5-glutathionyl-6-hydroxy-5,6-dihydronaphthalene	0.0188	1.2724	up	Metabolism of xenobiotics by cytochrome P450(ko00980)

	97174	63483		
5-Aminopentanal	0.001763568	1.522773847	up	Lysine degradation(ko00310)
6-Acetamido-2-oxohexanoate	0.000255744	1.602909414	up	Lysine degradation(ko00310)
D-Lysopine	0.015446424	1.32406041	up	Lysine degradation(ko00310)
Pyruvic acid	0.021871529	1.177006483	up	Insulin resistance(ko04931);;Cysteine and methionine metabolism(ko00270);;C5-Branched dibasic acid metabolism(ko00660);;Alanine, aspartate and glutamate metabolism(ko00250);;Phosphonate and phosphinate metabolism(ko00440);;Taurine and hypotaurine metabolism(ko00430);;Pyruvate metabolism(ko00620);;Pantothenate and CoA biosynthesis(ko00770);;Butanoate metabolism(ko00650);;Pentose and glucuronate interconversions(ko00040);;Glucagon signaling pathway(ko04922);;Glyoxylate and dicarboxylate metabolism(ko00630);;Thiamine metabolism(ko00730);;D-Amino acid metabolism(ko00470);;HIF-1 signaling pathway(ko04066);;Diabetic cardiomyopathy(ko05415);;Nicotinate and nicotinamide metabolism(ko00760);;Type II diabetes mellitus(ko04930);;Glycine, serine and threonine metabolism(ko00260);;Glycolysis / Gluconeogenesis(ko00010);;Phenylalanine

				metabolism(ko00360);;Valine, leucine and isoleucine biosynthesis(ko00290);;Tyrosine metabolism(ko00350);;Insulin secretion(ko04911);;Central carbon metabolism in cancer(ko05230);;Terpenoid backbone biosynthesis(ko00900);;Monobactam biosynthesis(ko00261);;Ascorbate and aldarate metabolism(ko00053);;AMPK signaling pathway(ko04152);;Pentose phosphate pathway(ko00030);;Arginine and proline metabolism(ko00330);;Citrate cycle (TCA cycle)(ko00020)
1-(5-Phosphoribosyl)imidazole-4-acetate	0.026862662	1.255799707	up	Histidine metabolism(ko00340)
4-(beta-Acetylaminoethyl)imidazole	0.040648493	1.197159623	up	Histidine metabolism(ko00340)
Arbutin	0.016293652	1.269515202	up	Glycolysis / Gluconeogenesis(ko00010)
Trypanothione disulfide	0.01300398	1.291090606	up	Glutathione metabolism(ko00480)
Trypanothione	0.0062552	1.3448064	up	Glutathione metabolism(ko00480)

	01	45		
Stachyose	0.0<br>243<br>951<br>74	1.2<br>564<br>573<br>86	up	Galactose metabolism(ko00052)
L-Fucose 1-phosphate	0.0<br>031<br>732<br>69	1.5<br>727<br>601<br>04	up	Fructose and mannose metabolism(ko00051);;Amino sugar and nucleotide sugar metabolism(ko00520)
L-Rhamnulose	0.0<br>010<br>652<br>08	1.5<br>544<br>194<br>91	up	Fructose and mannose metabolism(ko00051)
trans-Tetradec-2-enoyl-CoA	0.0<br>065<br>180<br>8	1.4<br>243<br>082<br>23	up	Fatty acid elongation(ko00062);;Fatty acid degradation(ko00071)
2-trans-Dodecenoyl-CoA	0.0<br>191<br>840<br>39	1.1<br>864<br>270<br>41	up	Fatty acid elongation(ko00062);;Fatty acid degradation(ko00071)
5'-Deoxy-5-fluorocytidine	0.0<br>007<br>415<br>7	1.6<br>281<br>360<br>51	up	Drug metabolism - other enzymes(ko00983)
floxuridine	0.0	1.1	up	Drug metabolism - other enzymes(ko00983)

	22028246	90146503		
Isonicotinoyl-NAD adduct	0.004964835	1.392701505	up	Drug metabolism - other enzymes(ko00983)
SN-38	0.018794152	1.289739833	up	Drug metabolism - other enzymes(ko00983)
NADP+	0.001932896	1.521565297	up	Diabetic cardiomyopathy(ko05415);;Nicotinate and nicotinamide metabolism(ko00760);;Alcoholic liver disease(ko04936);;Drug metabolism - other enzymes(ko00983);;Chemical carcinogenesis - reactive oxygen species(ko05208);;Aldosterone synthesis and secretion(ko04925);;Glutathione metabolism(ko00480);;Thyroid hormone synthesis(ko04918)
Linatine	0.019465501	1.29452308	up	D-Amino acid metabolism(ko00470)
S-Adenosylhomocysteine	0.023780109	1.254343503	up	Cysteine and methionine metabolism(ko00270);;Chemical carcinogenesis - reactive oxygen species(ko05208)
S-Adenosylmethioninamine	0.0009	1.5030	up	Cysteine and methionine metabolism(ko00270);;Arginine and proline metabolism(ko00330)

	40595	87169		
5'-Methylthioadenosine	9.55E-05	1.609511728	up	Cysteine and methionine metabolism(ko00270)
16-Oxopalmitate	0.031852734	1.240261224	up	Cutin, suberine and wax biosynthesis(ko00073)
Angiotensin (1-7)	0.004063671	1.418093594	up	Coronavirus disease - COVID-19(ko05171);;Renin-angiotensin system(ko04614)
Bradykinin	0.002563712	1.423450575	up	Complement and coagulation cascades(ko04610)
Glycocholate	0.013409862	1.262250636	up	Cholesterol metabolism(ko04979);;Primary bile acid biosynthesis(ko00120);;Bile secretion(ko04976)
Benzene	0.021125828	1.234380381	up	Chemical carcinogenesis - reactive oxygen species(ko05208)

7-Hydroxymethyl-12-methylbenz[a]anthracene	0.002159246	1.490247244	up	Chemical carcinogenesis - DNA adducts(ko05204);;Metabolism of xenobiotics by cytochrome P450(ko00980)
L-Proline	0.002780714	1.431943168	up	Central carbon metabolism in cancer(ko05230);;Aminoacyl-tRNA biosynthesis(ko00970);;Arginine and proline metabolism(ko00330);;ABC transporters(ko02010);;Protein digestion and absorption(ko04974);;D-Amino acid metabolism(ko00470);;Mineral absorption(ko04978)
3,6,8-Trimethylallantoin	0.00556272	1.372060837	up	Caffeine metabolism(ko00232)
2-Hydroxyparaconate	0.001577839	1.506759983	up	C5-Branched dibasic acid metabolism(ko00660)
Docosenoyl-CoA	0.020127375	1.333455923	up	Biosynthesis of unsaturated fatty acids(ko01040)
Montanoyl-CoA	0.024789589	1.309343649	up	Biosynthesis of unsaturated fatty acids(ko01040)
Docosanoyl-CoA	0.0235	1.3002	up	Biosynthesis of unsaturated fatty acids(ko01040)

	87507	95416		
(13Z,16Z)-Docosadienoic acid	0.002552622	1.476943035	up	Biosynthesis of unsaturated fatty acids(ko01040)
Levofloxacin	0.021030019	1.301402572	up	Bile secretion(ko04976)
BQ 123	9.50E-05	1.667670632	up	Bile secretion(ko04976)
Zalcitabine	0.020117537	1.348810257	up	Bile secretion(ko04976)
Tetracycline	0.016043242	1.254520897	up	Bile secretion(ko04976)
Vinblastine	0.008630601	1.309377207	up	Bile secretion(ko04976)

Digoxin	0.0003018 66	1.6330379 13	up	Bile secretion(ko04976)
Pantothenic acid	0.0063376 77	1.4629190 17	up	beta-Alanine metabolism(ko00410);;Vitamin digestion and absorption(ko04977);;Pantothenate and CoA biosynthesis(ko00770)
Serotonin	0.0103082 81	1.2796066 88	up	Axon regeneration(ko04361);;Tryptophan metabolism(ko00380);;Serotonergic synapse(ko04726);;Gap junction(ko04540);;Bile secretion(ko04976);;Taste transduction(ko04742);;Neuroactive ligand-receptor interaction(ko04080);;Inflammatory mediator regulation of TRP channels(ko04750);;Synaptic vesicle cycle(ko04721);;Chemical carcinogenesis - receptor activation(ko05207);;cAMP signaling pathway(ko04024)
L-Ascorbic acid	0.0359965 69	1.0991875 31	up	Ascorbate and aldarate metabolism(ko00053);;Glutathione metabolism(ko00480);;Vitamin digestion and absorption(ko04977);;HIF-1 signaling pathway(ko04066)
Ascorbic acid	0.0039913 87	1.3911375 8	up	Ascorbate and aldarate metabolism(ko00053);;Glutathione metabolism(ko00480);;Vitamin digestion and absorption(ko04977);;HIF-1 signaling pathway(ko04066)
D-Octopine	0.0042024 91	1.4052846 96	up	Arginine and proline metabolism(ko00330);;ABC transporters(ko02010)

CMP-pseudaminic acid	0.029734297	1.212042196	up	Amino sugar and nucleotide sugar metabolism(ko00520)
Undecaprenyl phosphate alpha-L-Ara4N	0.020803559	1.262158042	up	Amino sugar and nucleotide sugar metabolism(ko00520)
Pseudaminic acid	0.031589062	1.260825468	up	Amino sugar and nucleotide sugar metabolism(ko00520)
OPC8-CoA	0.032195975	1.251098078	up	alpha-Linolenic acid metabolism(ko00592)
D-Glucosamine 6-phosphate	0.009188429	1.427059594	up	Alanine, aspartate and glutamate metabolism(ko00250);;Amino sugar and nucleotide sugar metabolism(ko00520);;Diabetic cardiomyopathy(ko05415);;Insulin resistance(ko04931)
L-Isoleucine	0.001740369	1.460843973	up	ABC transporters(ko02010);;Valine, leucine and isoleucine biosynthesis(ko00290);;Mineral absorption(ko04978);;Valine, leucine and isoleucine degradation(ko00280);;Shigellosis(ko05131);;Central carbon metabolism in cancer(ko05230);;Protein digestion and absorption(ko04974);;Aminoacyl-tRNA biosynthesis(ko00970)
4-Amino-5-hydroxymethyl-2-meth	0.0	1.1	up	ABC transporters(ko02010);;Thiamine metabolism(ko00730)

ylpyrimidine	38490975	08968741		
Cytidine	0.003205235	1.432153919	up	ABC transporters(ko02010);;Pyrimidine metabolism(ko00240)
Maltotriose	0.01614197	1.367882667	up	ABC transporters(ko02010);;Carbohydrate digestion and absorption(ko04973)
Nopaline	0.001408756	1.49125226	up	ABC transporters(ko02010);;Arginine and proline metabolism(ko00330)
Isomaltotriose	0.02413296	1.245279171	up	ABC transporters(ko02010)
alpha-1,5-L-Arabinotriose	0.027919473	1.224943937	up	ABC transporters(ko02010)
