# Supplementary material for: Preliminary Exploration of the Protective Mechanism of Eugenol Against Acute Liver Injury Induced by Thioacetamide Based on Metabolomics
Source: Molecules. 2024 Nov 8;29(22):5288. doi: 10.3390/molecules29225288 (PMC11596261; doi:10.3390/molecules29225288)
Supplement: Supplementary file 1 [file molecules-29-05288-s001.zip › Supplementary Table S1 Differential expressed metab-final.pdf]

Supplementary Table S1. Differential expressed metabolites between the TAA group and control group.

name	Pvalue	VIP	regulated	KEGG_pathway_annotation
4-Hydroxy-L-threonine	0.045529706	1.3658875	up	Vitamin B6 metabolism(ko00750)
L-Isoleucine	0.013236436	1.604807518	down	Valine, leucine and isoleucine degradation(ko00280);;Valine, leucine and isoleucine biosynthesis(ko00290);;Aminoacyl-tRNA biosynthesis(ko00970);;Metabolic pathways(ko01100);;2-Oxocarboxylic acid metabolism(ko01210);;Biosynthesis of amino acids(ko01230);;ABC transporters(ko02010);;Protein digestion and absorption(ko04974);;Mineral absorption(ko04978);;Shigellosis(ko05131);;Central carbon metabolism in cancer(ko05230)
Vitamin K1	0.002172141	1.931084606	up	Ubiquinone and other terpenoid-quinone biosynthesis(ko00130);;Metabolic pathways(ko01100);;Biosynthesis of cofactors(ko01240);;Vitamin digestion and absorption(ko04977)
2-Phytyl-1,4-naphthoquinone	0.005212857	1.992606414	up	Ubiquinone and other terpenoid-quinone biosynthesis(ko00130);;Metabolic pathways(ko01100);;Biosynthesis of cofactors(ko01240)
alpha-Tocotrienol	0.0	1.5	do	Ubiquinone and other terpenoid-quinone biosynthesis(ko00130);;Metabolic

	38721675	47639881	wn	pathways(ko01100)
5,6-Indolequinone-2-carboxylic acid	0.016076775	1.758826123	up	Tyrosine metabolism(ko00350);;Metabolic pathways(ko01100)
5-Hydroxy-L-tryptophan	0.009236034	1.6700552	up	Tryptophan metabolism(ko00380);;Metabolic pathways(ko01100);;Axon regeneration(ko04361);;Serotonergic synapse(ko04726)
4-Hydroxy-2-quinolinecarboxylic acid	0.045973343	1.419699591	down	Tryptophan metabolism(ko00380);;Metabolic pathways(ko01100)
4-Amino-5-hydroxymethyl-2-methylpyrimidine	0.00030184	2.085335999	down	Thiamine metabolism(ko00730);;Metabolic pathways(ko01100);;Biosynthesis of cofactors(ko01240);;ABC transporters(ko02010)
2-(2-Carboxy-4-methylthiazol-5-yl)ethyl phosphate	0.027649934	1.593505246	down	Thiamine metabolism(ko00730);;Metabolic pathways(ko01100);;Biosynthesis of cofactors(ko01240)
(R)-Mevalonate	0.0093102	1.7335846	down	Terpenoid backbone biosynthesis(ko00900);;Metabolic pathways(ko01100);;Ferroptosis(ko04216)

	4	44		
Isopentenyl phosphate	0.000810621	1.95573066	down	Terpenoid backbone biosynthesis(ko00900);;Metabolic pathways(ko01100)
Testosterone	0.021213965	1.608749786	up	Steroid hormone biosynthesis(ko00140);;Metabolic pathways(ko01100);;Endocrine resistance(ko01522);;Ovarian steroidogenesis(ko04913);;GnRH secretion(ko04929);;Pathways in cancer(ko05200);;Prostate cancer(ko05215)
7alpha-Hydroxydehydroepiandrosterone	0.029797586	1.581026199	up	Steroid hormone biosynthesis(ko00140)
2-Methoxyestrone	0.042683687	1.516608327	up	Steroid hormone biosynthesis(ko00140)
Allopregnanolone	0.034188542	1.490429115	up	Steroid hormone biosynthesis(ko00140)
Cortolone	0.012283231	1.766317401	up	Steroid hormone biosynthesis(ko00140)
4alpha-Methylzymostero	0.0	1.6	do	Steroid biosynthesis(ko00100);;Metabolic pathways(ko01100)

l-4-carboxylate	25653043	74212512	wn	
Maltose	0.042113585	1.496612362	down	Starch and sucrose metabolism(ko00500);;Metabolic pathways(ko01100);;ABC transporters(ko02010);;Taste transduction(ko04742);;Carbohydrate digestion and absorption(ko04973)
Cellobiose	0.032352626	1.536705118	down	Starch and sucrose metabolism(ko00500);;Metabolic pathways(ko01100);;ABC transporters(ko02010)
Sphingosyl-phosphocholine	0.045432359	1.417871027	up	Sphingolipid metabolism(ko00600)
Retinol	0.017957939	1.748484033	down	Retinol metabolism(ko00830);;Metabolic pathways(ko01100);;Biosynthesis of cofactors(ko01240);;Vitamin digestion and absorption(ko04977)
(R)-3-Amino-2-methylpropanoate	0.028718441	1.550356814	up	Pyrimidine metabolism(ko00240);;Valine, leucine and isoleucine degradation(ko00280);;Metabolic pathways(ko01100)
Deoxycytidine	0.0090067	1.6617681	down	Pyrimidine metabolism(ko00240);;Metabolic pathways(ko01100);;Nucleotide metabolism(ko01232);;ABC transporters(ko02010)

	43	96		
Cytosine	0.048874503	1.447702551	down	Pyrimidine metabolism(ko00240);;Metabolic pathways(ko01100);;Nucleotide metabolism(ko01232)
GTP	0.033019611	1.471916616	down	Purine metabolism(ko00230);;Riboflavin metabolism(ko00740);;Folate biosynthesis(ko00790);;Metabolic pathways(ko01100);;Nucleotide metabolism(ko01232);;Biosynthesis of cofactors(ko01240);;Ras signaling pathway(ko04014);;Rap1 signaling pathway(ko04015);;Sulfur relay system(ko04122);;Autophagy - animal(ko04140);;Endocytosis(ko04144)
Deoxyguanosine	0.019894032	1.753481504	up	Purine metabolism(ko00230);;Metabolic pathways(ko01100);;Nucleotide metabolism(ko01232);;ABC transporters(ko02010)
dAMP	0.016635858	1.613385601	down	Purine metabolism(ko00230);;Metabolic pathways(ko01100);;Nucleotide metabolism(ko01232)
Deoxyadenosine monophosphate	0.044020497	1.397517301	up	Purine metabolism(ko00230);;Metabolic pathways(ko01100);;Nucleotide metabolism(ko01232)
1-(5-Phospho-D-ribosyl)-5-amino-4-imidazolecarboxylate	0.026928014	1.472968335	up	Purine metabolism(ko00230);;Metabolic pathways(ko01100)

3alpha,7alpha,12alpha,26-Tetrahydroxy-5beta-cholestane	0.0065453 62	1.8814508 06	up	Primary bile acid biosynthesis(ko00120);;Metabolic pathways(ko01100)
3alpha,7alpha,12alpha-Trihydroxy-5beta-cholestane	0.0343630 95	1.4771489 53	up	Primary bile acid biosynthesis(ko00120);;Metabolic pathways(ko01100)
3beta,7alpha-Dihydroxy-5-cholestenoate	0.0370332 51	1.4143600 62	up	Primary bile acid biosynthesis(ko00120)
7alpha-Hydroxy-3-oxo-4-cholestenoate	1.41E-05	2.2752702 24	up	Primary bile acid biosynthesis(ko00120)
Biliverdin	0.0361304 86	1.5699702 08	down	Porphyrin metabolism(ko00860);;Metabolic pathways(ko01100)
D-Glucosaminate-6-phosphate	0.0376439 45	1.4260929 39	down	Pentose phosphate pathway(ko00030)
Saccharopine	0.0231	1.4851	down	Lysine biosynthesis(ko00300);;Lysine degradation(ko00310);;Metabolic pathways(ko01100);;Biosynthesis of amino acids(ko01230)

	92868	41569		
L-Lactic acid	0.032623002	1.556636203	down	Glycolysis / Gluconeogenesis(ko00010);;Fructose and mannose metabolism(ko00051);;Pyruvate metabolism(ko00620);;Propanoate metabolism(ko00640);;Metabolic pathways(ko01100);;cAMP signaling pathway(ko04024);;HIF-1 signaling pathway(ko04066);;Glucagon signaling pathway(ko04922);;Central carbon metabolism in cancer(ko05230)
Phosphocholine	0.007012193	1.82062035	down	Glycerophospholipid metabolism(ko00564);;Metabolic pathways(ko01100);;Choline metabolism in cancer(ko05231)
Glycerophosphocholine	0.042259701	1.504878058	down	Glycerophospholipid metabolism(ko00564);;Ether lipid metabolism(ko00565);;Choline metabolism in cancer(ko05231)
CDP-glycerol	0.007152629	1.79351754	up	Glycerophospholipid metabolism(ko00564)
Oxidized glutathione	0.044160655	1.489850474	up	Glutathione metabolism(ko00480);;Metabolic pathways(ko01100);;Biosynthesis of cofactors(ko01240);;Ferroptosis(ko04216);;Thyroid hormone synthesis(ko04918);;Chemical carcinogenesis - reactive oxygen species(ko05208);;Diabetic cardiomyopathy(ko05415)
Trypanothione	0.0129130	1.6410259	down	Glutathione metabolism(ko00480);;Metabolic pathways(ko01100)

	31	48		
D-Fructose 6-phosphate	0.040893839	1.423973715	down	Galactose metabolism(ko00052);;Starch and sucrose metabolism(ko00500);;Metabolic pathways(ko01100);;Carbon metabolism(ko01200);;AMPK signaling pathway(ko04152);;Glucagon signaling pathway(ko04922);;Insulin resistance(ko04931);;Central carbon metabolism in cancer(ko05230);;Diabetic cardiomyopathy(ko05415)
D-Galactose	0.006958231	1.788153601	down	Galactose metabolism(ko00052);;Metabolic pathways(ko01100);;Carbohydrate digestion and absorption(ko04973);;Mineral absorption(ko04978)
Stachyose	0.045833803	1.588879833	down	Galactose metabolism(ko00052);;Metabolic pathways(ko01100)
L-Sorbose	0.0467072	1.418095008	down	Fructose and mannose metabolism(ko00051);;Metabolic pathways(ko01100)
L-Rhamnulose	0.016339712	1.710823828	down	Fructose and mannose metabolism(ko00051);;Metabolic pathways(ko01100)
Biopterin	0.020096278	1.577073757	down	Folate biosynthesis(ko00790);;Metabolic pathways(ko01100);;Biosynthesis of cofactors(ko01240)

Sepiapterin	0.009540572	1.80270539	up	Folate biosynthesis(ko00790);;Metabolic pathways(ko01100)
1-Octadecanoyl-2-(7Z,10Z,13Z,16Z-docosatetraenoyl)-sn-glycero-3-phosphoethanolamine	0.038687096	1.476154799	up	Ferroptosis(ko04216)
1-Octadecanoyl-sn-glycero-3-phosphoethanolamine	0.021349604	1.555257464	up	Ferroptosis(ko04216)
2-trans-Dodecenoyl-CoA	0.02458882	1.560856777	down	Fatty acid elongation(ko00062);;Fatty acid degradation(ko00071);;Metabolic pathways(ko01100);;Fatty acid metabolism(ko01212)
trans,cis-Lauro-2,6-dienoyl-CoA	0.006425266	1.749782814	up	Fatty acid degradation(ko00071)
Decanoic acid	0.028310226	1.615077818	up	Fatty acid biosynthesis(ko00061);;Metabolic pathways(ko01100)
Caprylic acid	0.0100	1.8667	up	Fatty acid biosynthesis(ko00061);;Lipoic acid metabolism(ko00785);;Metabolic pathways(ko01100);;Biosynthesis of cofactors(ko01240)

	92888	13087		
D-Ornithine	0.046394072	1.326098116	down	D-Amino acid metabolism(ko00470);;Metabolic pathways(ko01100)
O-Succinyl-L-homoserine	0.004832076	1.942430784	up	Cysteine and methionine metabolism(ko00270);;Sulfur metabolism(ko00920);;Metabolic pathways(ko01100);;Biosynthesis of amino acids(ko01230)
S-Adenosylhomocysteine	0.04746778	1.540319066	down	Cysteine and methionine metabolism(ko00270);;Metabolic pathways(ko01100);;Biosynthesis of amino acids(ko01230);;Biosynthesis of cofactors(ko01240);;Chemical carcinogenesis - reactive oxygen species(ko05208)
Cyclic ADP-ribose	0.024367514	1.530779978	up	Calcium signaling pathway(ko04020);;Oxytocin signaling pathway(ko04921);;Salivary secretion(ko04970);;Pancreatic secretion(ko04972)
Biotin sulfoxide	0.013833178	1.707491994	up	Biotin metabolism(ko00780);;Metabolic pathways(ko01100)
Adrenic acid	0.011444462	1.77904255	up	Biosynthesis of unsaturated fatty acids(ko01040);;Ferroptosis(ko04216)

Docosenoyl-CoA	0.040052815	1.587083664	down	Biosynthesis of unsaturated fatty acids(ko01040);;Fatty acid metabolism(ko01212)
Montanoyl-CoA	0.020531545	1.776824359	down	Biosynthesis of unsaturated fatty acids(ko01040);;Fatty acid metabolism(ko01212)
Docosanoyl-CoA	0.029214423	1.684650776	down	Biosynthesis of unsaturated fatty acids(ko01040);;Fatty acid metabolism(ko01212)
Indinavir	0.010057483	1.685053646	down	Bile secretion(ko04976)
Ouabain	0.02065141	1.724475879	down	Bile secretion(ko04976)
Threonate	0.017037933	1.78296633	up	Ascorbate and aldarate metabolism(ko00053);;Metabolic pathways(ko01100)
Ascorbic acid	0.0385	1.4099	down	Ascorbate and aldarate metabolism(ko00053);;Glutathione metabolism(ko00480);;Metabolic pathways(ko01100);;Biosynthesis of

	48813	90067		cofactors(ko01240);;HIF-1 signaling pathway(ko04066);;Vitamin digestion and absorption(ko04977)
D-Octopine	0.005296462	1.781440117	down	Arginine and proline metabolism(ko00330);;Metabolic pathways(ko01100);;ABC transporters(ko02010)
L-4-Hydroxyglutamate semialdehyde	0.027362412	1.489700421	up	Arginine and proline metabolism(ko00330);;Metabolic pathways(ko01100)
20-HETE	0.022431441	1.750745546	down	Arachidonic acid metabolism(ko00590);;Metabolic pathways(ko01100);;Vascular smooth muscle contraction(ko04270)
Prostaglandin D2	6.38E-06	2.314755158	up	Arachidonic acid metabolism(ko00590);;Metabolic pathways(ko01100);;Neuroactive ligand-receptor interaction(ko04080);;Fc epsilon RI signaling pathway(ko04664);;Serotonergic synapse(ko04726);;African trypanosomiasis(ko05143);;Asthma(ko05310)
(15S)-15-Hydroxy-5,8,11-cis-13-trans-eicosatetraenoate	0.031201437	1.528598552	up	Arachidonic acid metabolism(ko00590);;Metabolic pathways(ko01100);;Inflammatory mediator regulation of TRP channels(ko04750)
6-Keto-prostaglandin F1alpha	0.018870466	1.586708004	up	Arachidonic acid metabolism(ko00590);;Metabolic pathways(ko01100)

6-Keto-prostaglandin E1	0.007833633	1.871098623	up	Arachidonic acid metabolism(ko00590);;Metabolic pathways(ko01100)
9(S)-HETE	0.000127976	2.222981352	up	Arachidonic acid metabolism(ko00590)
20-COOH-Leukotriene B4	0.039642198	1.527390987	up	Arachidonic acid metabolism(ko00590)
12-Keto-leukotriene B4	0.001828931	2.054876205	up	Arachidonic acid metabolism(ko00590)
CMP-pseudaminic acid	0.048753101	1.573179	down	Amino sugar and nucleotide sugar metabolism(ko00520);;Metabolic pathways(ko01100);;Biosynthesis of nucleotide sugars(ko01250)
N-Acetylneuraminate	0.040529335	1.475282649	up	Amino sugar and nucleotide sugar metabolism(ko00520);;Metabolic pathways(ko01100);;Biosynthesis of nucleotide sugars(ko01250)
Pseudaminic acid	0.0321	1.6504	down	Amino sugar and nucleotide sugar metabolism(ko00520);;Metabolic pathways(ko01100);;Biosynthesis of nucleotide sugars(ko01250)

	87024	44132		
Methyl jasmonate	5.14E-05	2.206295094	up	alpha-Linolenic acid metabolism(ko00592)
Succinate semialdehyde	0.021226226	1.723493083	down	Alanine, aspartate and glutamate metabolism(ko00250);;Tyrosine metabolism(ko00350);;Butanoate metabolism(ko00650);;Vitamin B6 metabolism(ko00750);;Nicotinate and nicotinamide metabolism(ko00760);;Metabolic pathways(ko01100);;Carbon metabolism(ko01200)
Maltotriose	0.025747319	1.704678946	down	ABC transporters(ko02010);;Carbohydrate digestion and absorption(ko04973)
Isomaltotriose	0.040628897	1.588548407	down	ABC transporters(ko02010)
alpha-1,5-L-Arabinotriose	0.033332113	1.632870444	down	ABC transporters(ko02010)
